# Cytotoxic Potential of Marine-Derived Fungi Isolated from Sponges and Brown Algae of Mauritius

**DOI:** 10.3390/md24080289

**Published:** 2026-08-21

**Authors:** Jessica Mélanie Wong Chin, Annaelle Hip Kam, Rajesh Jeewon, Abdulwahed Fahad Alrefaei, Teeshan Bahorun, Daneshwar Puchooa, Neil O. Carragher, Vidushi S. Neergheen

**Affiliations:** 1 Biopharmaceutical Unit, Center for Biomedical and Biomaterials Research (CBBR), University of Mauritius, Reduit 80837, Mauritius; melanie.jessica@hotmail.com (J.M.W.C.); annaellehip@gmail.com (A.H.K.); 2 Department of Agricultural and Food Science, Faculty of Agriculture, University of Mauritius, Reduit 80837, Mauritius; daneshwarpuchooa@gmail.com; 3 Department of Health Sciences, Faculty of Medicine and Health Sciences, University of Mauritius, Reduit 80837, Mauritius; r.jeewon@uom.ac.mu; 4 Honghe Center for Mountain Futures, Kunming Institute of Botany, Chinese Academy of Sciences, Honghe 654400, China; 5 School of Pharmacy, Zunyi Medical University, Zunyi 563000, China; 6 Department of Zoology, College of Science, King Saud University, P.O. Box 2455, Riyadh 11451, Saudi Arabia; afrefaei@ksu.edu.sa; 7 Mauritius Research and Innovation Council, Ebene 72201, Mauritius; t.bahorun@mric.mu; 8 Cancer Research UK Scotland Centre, Institute of Genetics and Cancer, The University of Edinburgh, Edinburgh EH4 2XU, UK; n.carragher@ed.ac.uk

**Keywords:** marine, fungi, cytotoxic, metabolites, sponge, algae, fungal endophytes, Mauritius

## Abstract

Marine fungi associated with sponges and brown algae are promising sources of pharmacologically active compounds. This study investigated the cytotoxic potential and metabolomic profiles of fungal strains isolated from the marine environment of Mauritius. Among the twenty extracts screened, the mycelium extracts were more cytotoxic than the broth extracts. Four extracts demonstrated the most potent activity: *Aspergillus chevalieri* (F2M), *Aspergillus ochraceus* (F25M) and *Biatriospora* sp. (F34M, F34B). The algal endophyte *Aspergillus chevalieri* (F2M) mycelium extract displayed an IC_50_ of 14.27 ± 1.22 µg/mL after 24 h against HepG2 cells. The sponge-associated fungi *Aspergillus ochraceus* (F25M) showed promising cytotoxic activities against HepG2 cells (IC_50_ of 8.775 ± 0.78 µg/mL) after 24 h of treatment, with a selectivity index of 2.27, and had the lowest IC_50_ (2.49 ± 0.60 µg/mL after 24 h; 7.14 ± 3.14 µg/mL after 48 h) against FLO-1 cells, also reducing tumor spheroid growth and integrity during the first four hours. All four extracts increased the intracellular ROS production, but only the mycelium extract of *A. chevalieri* (F2M) significantly increased superoxide dismutase (SOD) and catalase (CAT) activity. Metabolomic profiling identified diverse compound classes, including alkaloids, terpenoids, amino acids, anthraquinones and coumarins. The findings revealed that the marine fungi from Mauritius are promising sources of cytotoxic metabolites that require purification and subsequent confirmation and mechanistic studies.

## 1. Introduction

Cancer is a serious global public health challenge. By 2050, the number of new cancer cases will rise to 33 million, and the number of cancer-related deaths will reach 18.2 million [1]. Despite significant advances in cancer treatment modalities, including targeted therapies, immunotherapy, and improved chemotherapeutic regimens, cancer continues to pose a major challenge, with incidence and mortality rates remaining high. Treatment resistance, disease recurrence, and toxicity continue to limit long-term clinical success across many cancer types. In this context, the exploration of novel sources of bioactive compounds remains highly desirable, both to expand the therapeutic arsenal and to identify agents with new mechanisms of action capable of overcoming existing limitations.

Marine organisms have made a substantial contribution to the development of anticancer drugs [2,3,4,5]. Drugs like cytarabine, eribulin mesylate, brentuximab vedotin, and trabectine are all marine-derived FDA-approved drugs which are used to treat leukemia, soft tissue sarcoma, and ovarian and breast cancer [6]. Marine fungi also produce chemically diverse molecules that have antimicrobial, antiviral, anti-inflammatory, antioxidant and anticancer properties [7,8,9,10]. Two new compounds, unguisol A and B, were obtained from the sponge-associated fungus *Aspergillus unguis*. They induced apoptosis in MDA-MB231 breast cancer cells, caused cell cycle arrest at the S phase and suppressed BCL2L1 and AKT1 mRNA expression [11]. The new compound corrallomycetellain J, obtained from the marine sediment fungus *Corallomycetella repens* HDN23-0007, showed potent cytotoxicity against ASPC-1 and NCI-H446/EP cell lines (IC_50_: 1.5–2.3 µM), surpassing the positive control Doxorubicin (IC_50_: 5.9–11.3 µM) [12]. Even though numerous anticancer metabolites have been reported from marine fungi, none of them have reached the market yet.

The Exclusive Economic Zone (EEZ) of Mauritius, which is about 2.3 million km^2^, harbors diverse sponges and algae, along with their associated fungi, representing a pool of bioactive metabolites. Macroalgae and sponges from the Mauritian waters have been investigated for their cytotoxic properties and hold immense potential in anticancer drug research [13,14,15,16]. Along with their hosts, the marine-derived fungi are also producers of cytotoxic compounds. For instance, Parthsarathy et al. [17] showed that the macroalgae fungal endophyte *Penicillium chrysogenum* was able to hinder the proliferation of MCF-7 breast cancer cells, with an IC_50_ value of 101.1 μg/mL, when grown in potato dextrose broth. Moreover, the marine-sponge-derived fungi *Ascomycota* sp. showed cytotoxicity against HepG2, MCF-7 and SKMel2 carcinoma cells, with IC_50_ ranging from 48.6–96.5 μM [18]. The distinctive ecosystem of the marine environment possibly enables a number of fungal microorganisms associated with marine sponges and macroalgae to produce compounds with potent biological activities. Taking these factors into consideration, marine fungi associated with brown algae and sponges in Mauritius are being studied for their cytotoxic potential.

This study investigates the cytotoxic potential and characterizations of bioactive compounds of marine-derived fungi extracted from sponges and brown algae in Mauritius, aiming to enhance the growing understanding of marine fungal bioactivity and facilitate the early-stage exploration of novel candidates for therapeutic development.

## 2. Results

### 2.1. Cytotoxicity of Fungal Extracts

Marine fungi were isolated from the selected sponge and brown algae species, as these hosts support highly diverse fungal communities. The hepatocellular carcinoma (HepG2), lung carcinoma (A549) and connective tissue liposarcoma (SW872) cells were included in this study to assess the cytotoxic potential of fungal extracts across different cancer cell types. The ten extracts (broth and mycelium), obtained from the five algae endophytes, showed cytotoxic activities against HepG2 and SW872 cells. Only eight extracts were able to inhibit the growth of A549 cells. The mycelium extract of *Aspergillus chevalieri* (F2M) had potent cytotoxicity against the three cancer cell lines included in this study. IC_50_ values of 14.27 ± 1.22 µg/mL, 29.66 ± 0.50 µg/mL and 25.43 ± 2.16 µg/mL were observed for HepG2 cells, A549 cells and SW872 cells, respectively (Table 1). It can also be noted that the extracts of *Aspergillus iizukae* (F37) displayed selective cytotoxicity against HepG2 cells. Overall, it can be observed that the IC_50_ was lower against HepG2 cells as compared to the other cell lines.

All the extracts from the sponge-associated fungi were able to inhibit the growth of HepG2 cells. Most of the extracts were also cytotoxic against A549 and SW872 cells, except for the broth extract of *Parengyodontium album* (F27B) (Table 2). The mycelium extract of *Aspergillus ochraceus* (F25M) had a low IC_50_ of 8.775 ± 0.78 µg/mL against HepG2 cells, an IC_50_ of 19.86 ± 1.46 µg/mL against A549 cells and an IC_50_ of 11.31 ± 1.18 µg/mL against SW872 cells. Another marine-derived fungi with cytotoxic properties against the three cancer cell lines used in this study was *Biatriospora* sp. Both the broth and mycelium extracts showed potent cytotoxicity. For the broth extract, an IC_50_ of 19.07 ± 1.70 µg/mL was observed for HepG2 cells, an IC_50_ of 22.85 ± 3.90 µg/mL was observed for A549 cells and an IC_50_ of 19.36 ± 2.36 µg/mL was observed against SW872 cells. Regarding the mycelium extract, an IC_50_ of 18.95 ± 1.49 µg/mL against HepG2 cells, an IC_50_ of 11.31 ± 4.75 µg/mL against A549 cells and an IC_50_ of 18.01 ± 1.96 µg/mL against SW872 cells were observed. Overall, the mycelium extracts were more cytotoxic compared to the broth extracts. There was a significant difference between the mean IC_50_ values of the mycelium and the broth extracts (*p* < 0.05).

**Figure 1:**
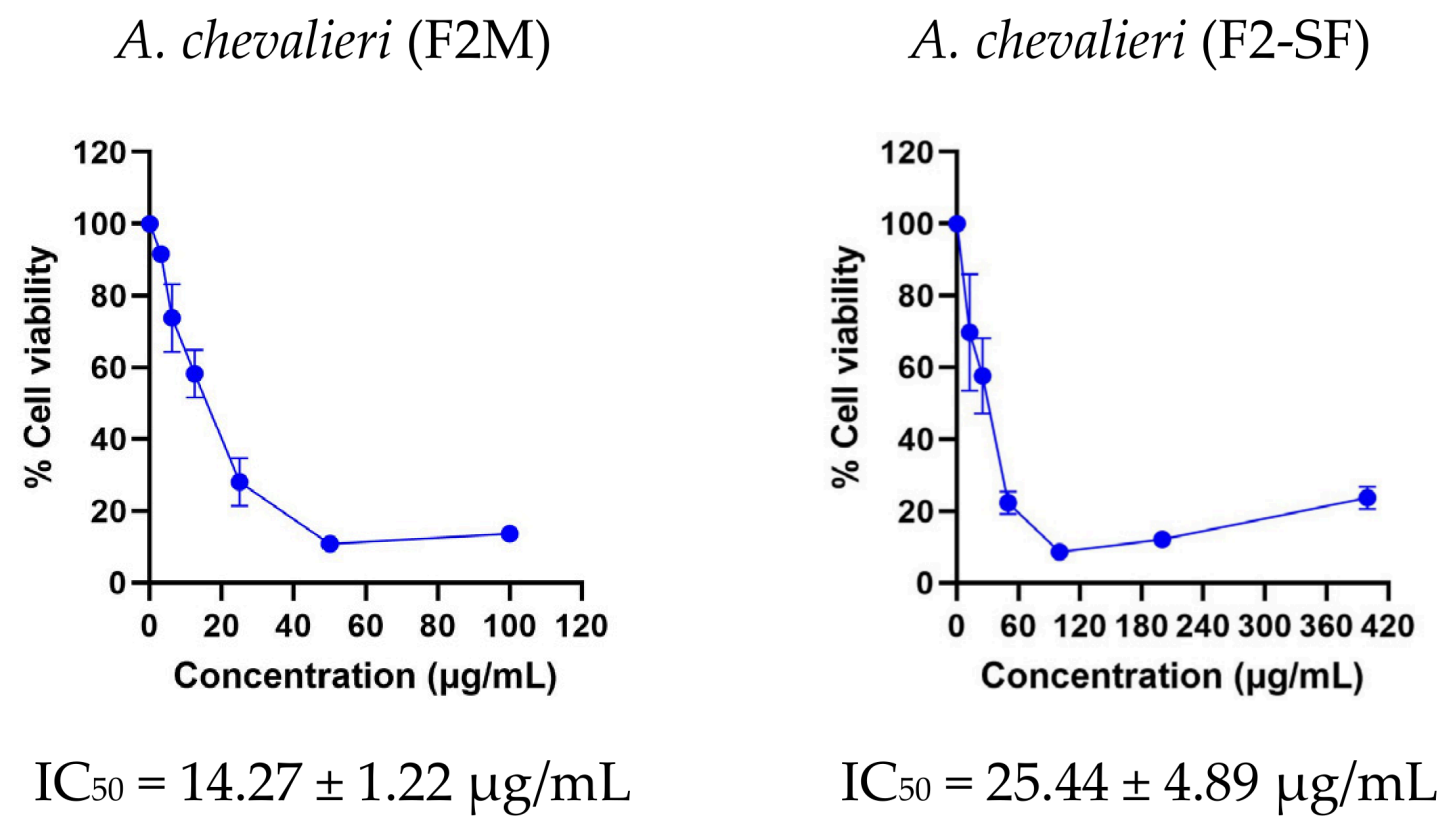

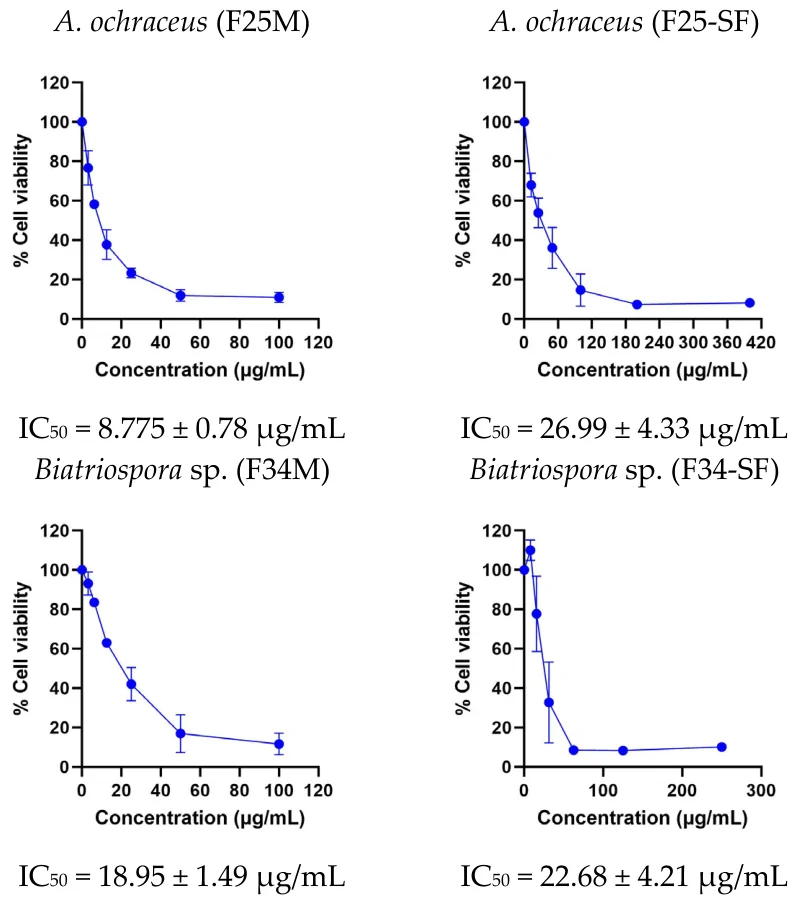
Percentage cell viability of liquid mycelium media and solid media fungal extracts. Data are presented as mean ± SD of three independent experiments, with each experiment performed in triplicate (*n* = 3).

A dose-dependent decrease in HepG2 cell viability was observed following treatment with the fungal extracts (Figure 1). Increasing concentration of the extract caused a decrease in the number of viable cancer cells. There was a significant difference between the mean IC_50_ values of the liquid media mycelium extract and the solid media extract (*p* < 0.05).

### 2.2. Esophageal Adenocarcinoma Cell Line and 3D Tumor Spheroid

The four fungal extracts selected in Section 2.1, based on their potent cytotoxic activity, were further evaluated against the esophageal cancer cell lines FLO-1 and OE33. All four extracts exhibited cytotoxic activity, as shown in Table 3. It can be noted that the extracts were more cytotoxic after 24 h, as compared to 48 h. There was a significant difference between the mean IC_50_ values obtained after the two time points (*p* < 0.05). The mycelium extract of *A. ochraceus* (F25M) had the lowest IC_50_ (2.49 ± 0.60 µg/mL after 24 h; 7.14 ± 3.14 µg/mL after 48 h) against FLO-1 cells. Regarding OE33 cells, the broth extract of *Biatriospora* sp. (F34B) was more cytotoxic after 24 h (IC_50_ 0.38 ± 0.37 µg/mL), while the mycelium extract of *A. ochraceus* (F25M) was more cytotoxic after 48 h (IC_50_ 3.14 ± 4.10 µg/mL).

**Figure 2:**
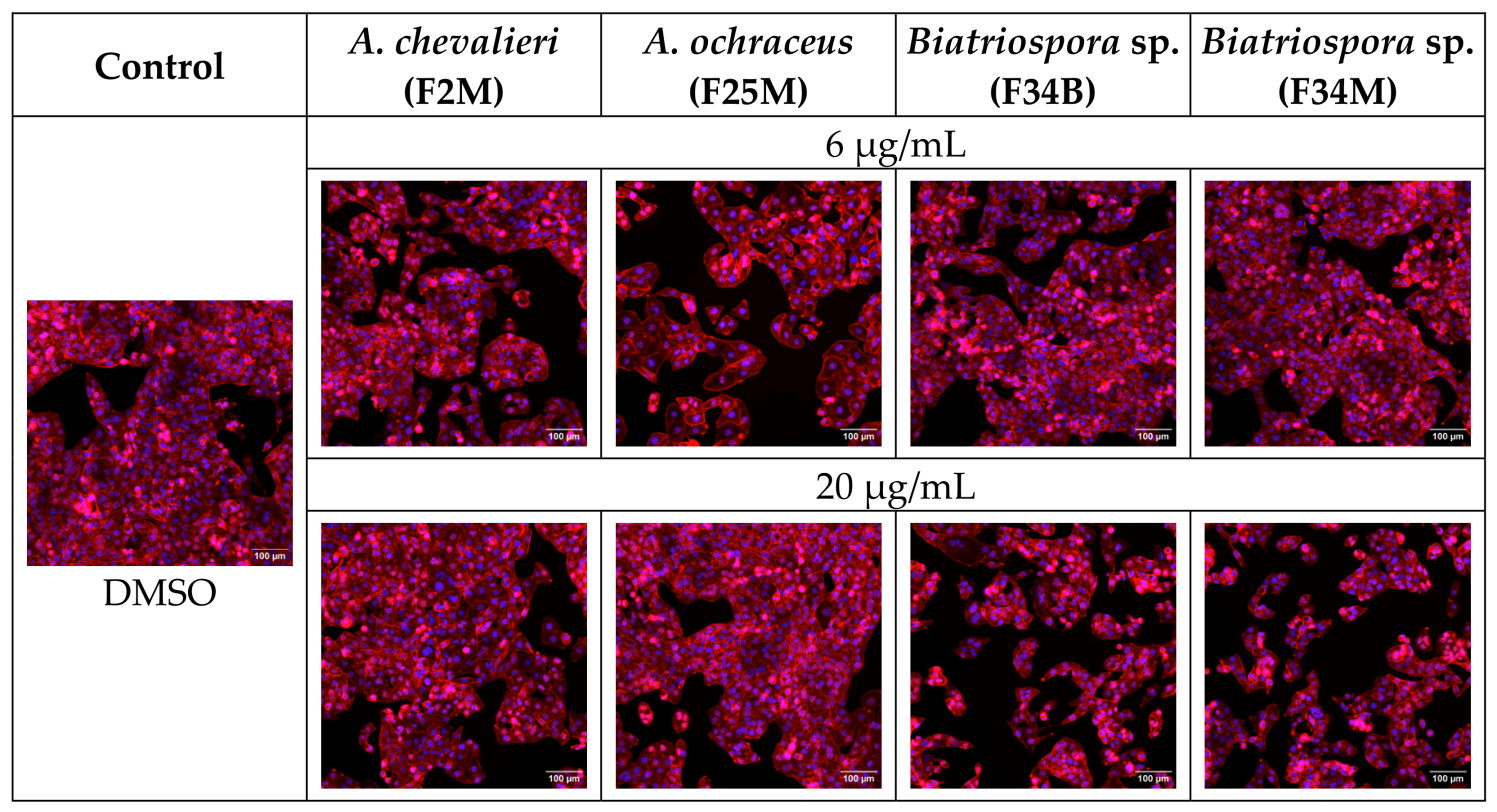
Fluorescence microscopy images showing cell count and morphological changes in the FLO-1 cells.

Morphological changes were observed when the FLO-1 cells were treated with the four extracts at different concentrations (Figure 2). A decrease in cell number and altered morphology resulting in cell shrinkage, detachment, and loss of structural integrity can be observed following treatment with 6 µg/mL of *A. chevalieri* (F2M) and *A. ochraceus* (F25M) and 20 µg/mL of *Biatriospora* sp. (F34B, F34M).

**Figure 3:**
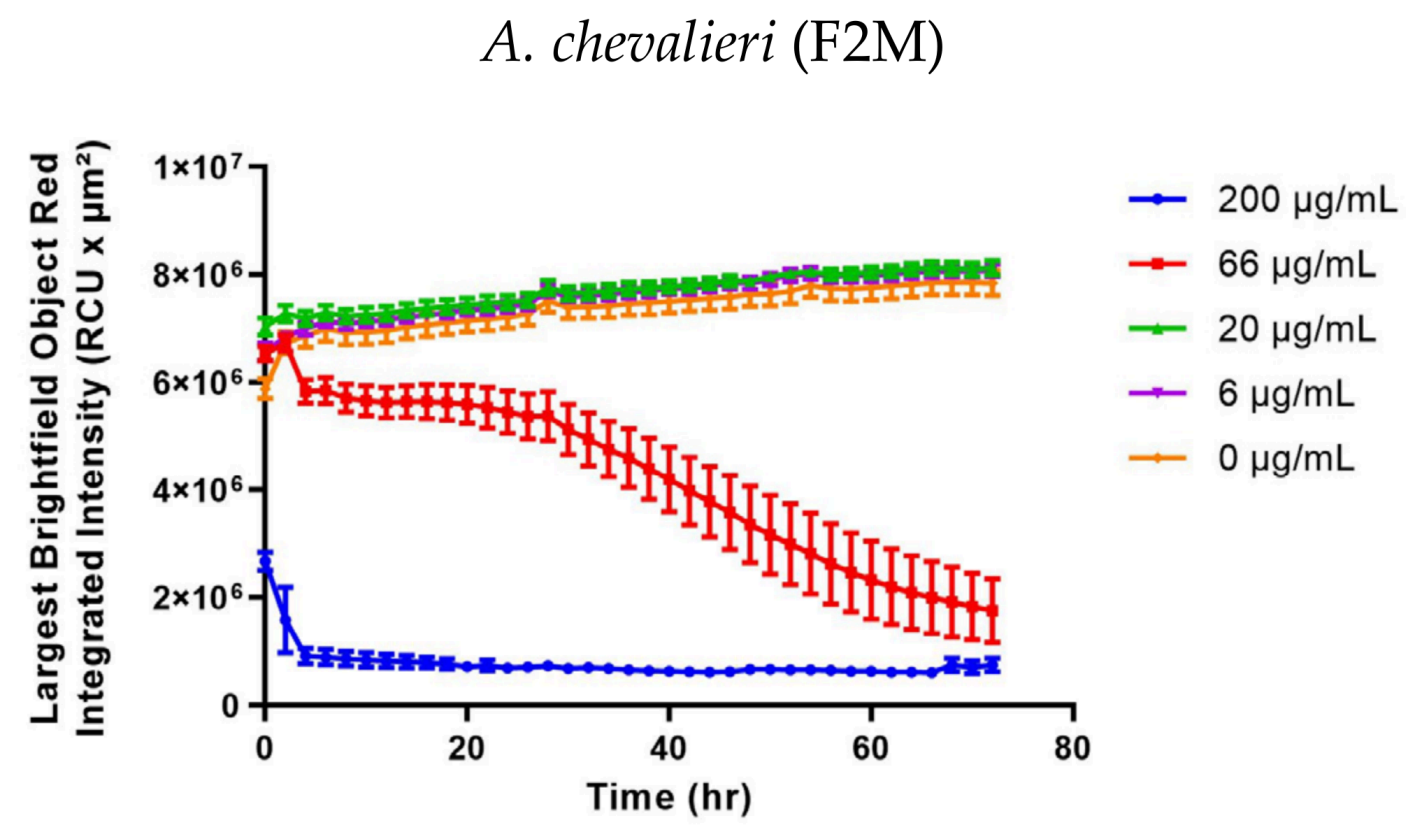

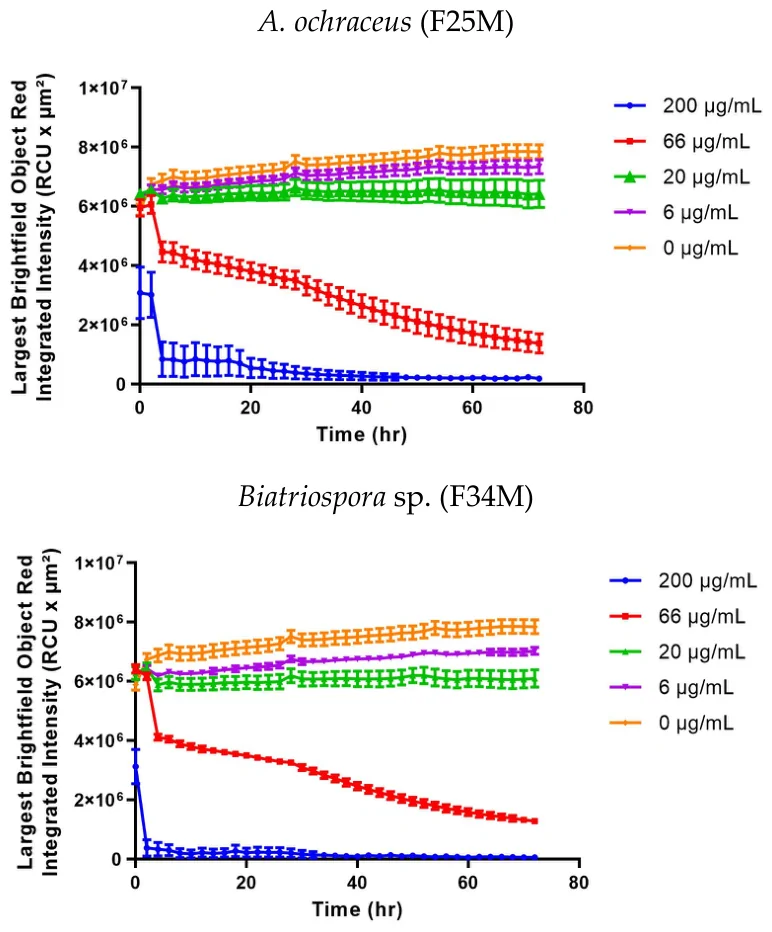
Effect of fungal extracts on FLO-1 spheroids monitored using the Incucyte-derived “Largest Brightfield Object Red Integrated Intensity” measurement over 72 h. Data are presented as mean ± SD of three independent experiments, with each experiment performed in triplicate (*n* = 3).

Figure 3 shows the effects of the three mycelium extracts, *A. chevalieri* (F2M), *A. ochraceus* (F25M) and *Biatriospora* sp. (F34M), on the Incucyte-derived image-based spheroid growth measurement at different concentrations. This was monitored using the ‘Largest Brightfield Object Red Integrated Intensity’ parameter, an image-based measurement used as a proxy for spheroid growth and integrity. A reduction in the Incucyte-derived spheroid-associated integrated intensity was observed at the highest concentrations of the extracts (200 µg/mL and 66 µg/mL) during the first four hours of exposure. A sustained inhibitory effect on spheroid growth was observed from after four hours until the end of the experiment. In contrast, treatment with lower concentrations (20 µg/mL and 6 µg/mL) did not result in significant change in the Incucyte-derived spheroid-associated integrated intensity.

The area under the curve (AUC) was calculated to quantify the overall response over the monitored time period. Treatment with the higher concentrations (200 µg/mL and 66 µg/mL) resulted in a significantly lower AUC than the untreated control, indicating a reduction in the Incucyte-derived spheroid-associated integrated intensity throughout the experiment (*p* < 0.05). In contrast, no significant differences in AUC were observed for the lower concentrations (20 µg/mL and 6 µg/mL), compared with the untreated control.

### 2.3. Intracellular ROS Production

**Figure 4:**
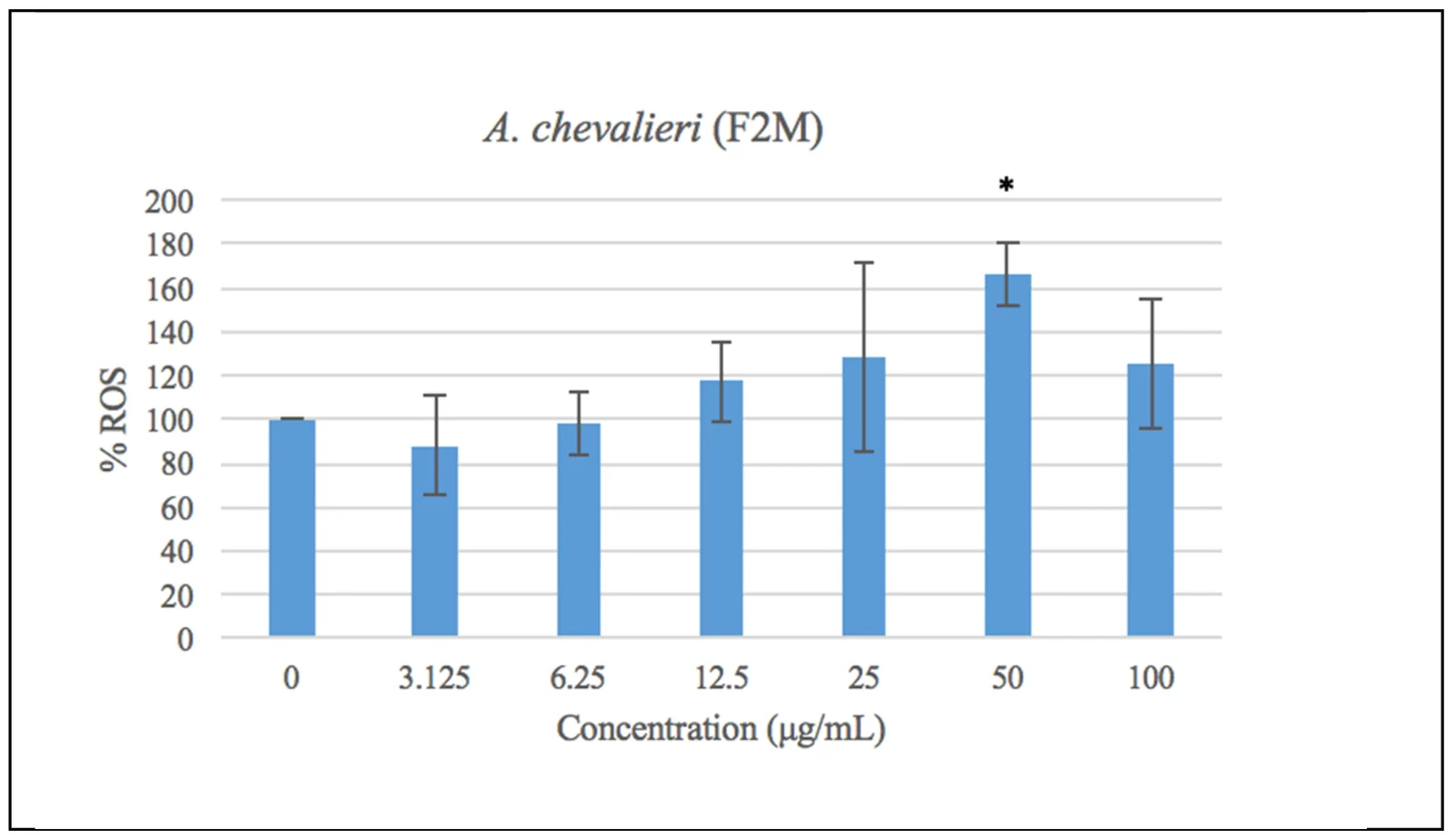

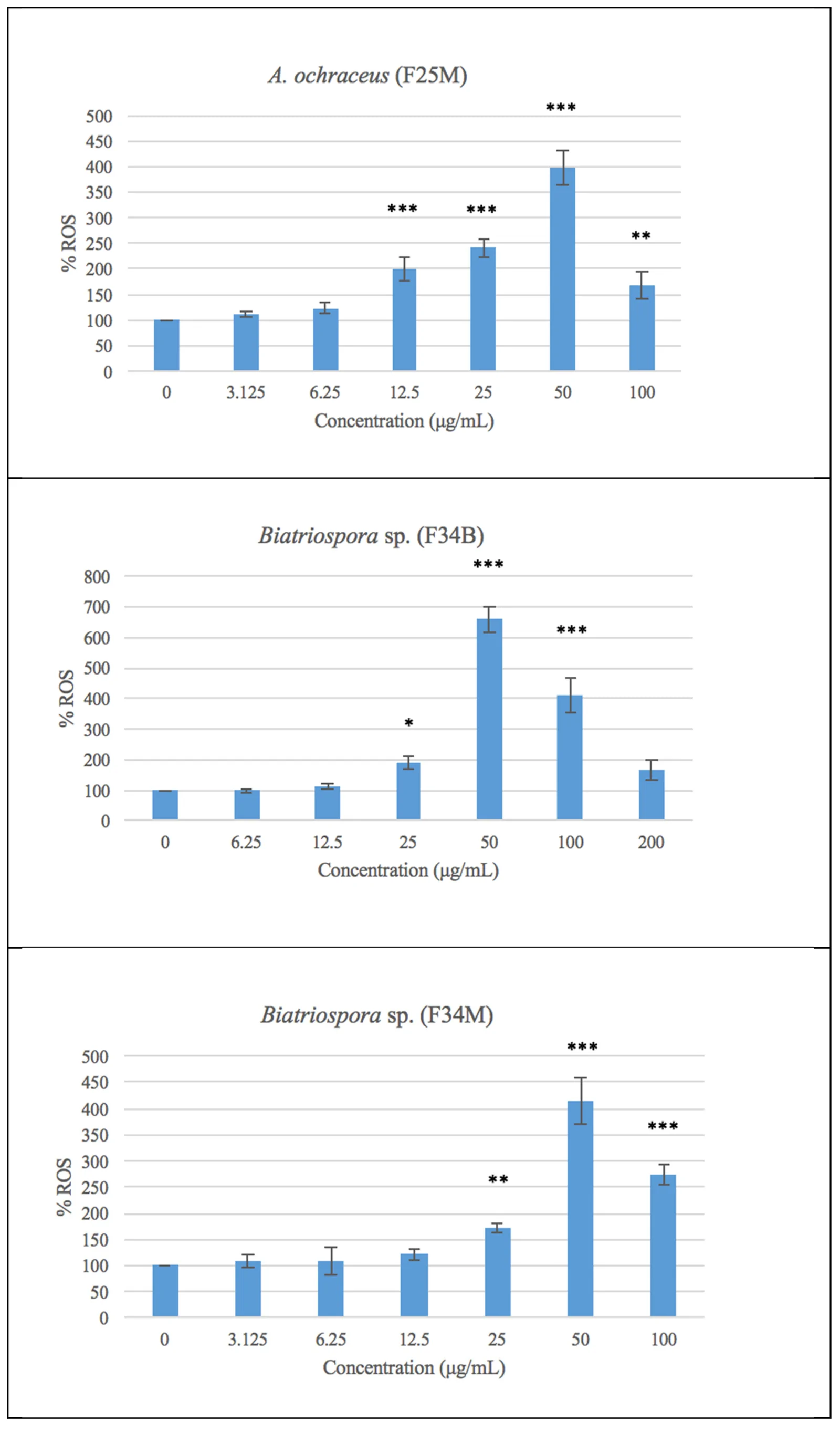
Modulation of intracellular ROS in HepG2 cells with different concentrations of extracts. Significance was assessed by one-way ANOVA followed by Dunnett’s test; * *p* < 0.05, ** *p* < 0.01, *** *p* < 0.001 (vs. control). Data are presented as mean ± SD of three independent experiments, with each experiment performed in triplicate (*n* = 3).

The four most active fungal extracts, selected based on their cytotoxic activity as reported in Section 2.1 and Section 2.2, were evaluated for their ability to modulate intracellular ROS production in order to investigate the potential mechanism underlying their cytotoxic effects. Modulation of intracellular ROS generation was assessed as a percentage of DCF-fluorescence. Treatment with the four extracts showed an upregulation in the production of cytosolic ROS in HepG2 cells, compared to the untreated control cells. ROS level increased significantly up to 50 µg/mL of extract (*p* < 0.05), followed by a decline at higher concentrations (Figure 4). It can be noted that ROS level peaked at 50 µg/mL for the four extracts.

### 2.4. Intrinsic Antioxidant Activities

**Figure 5:**
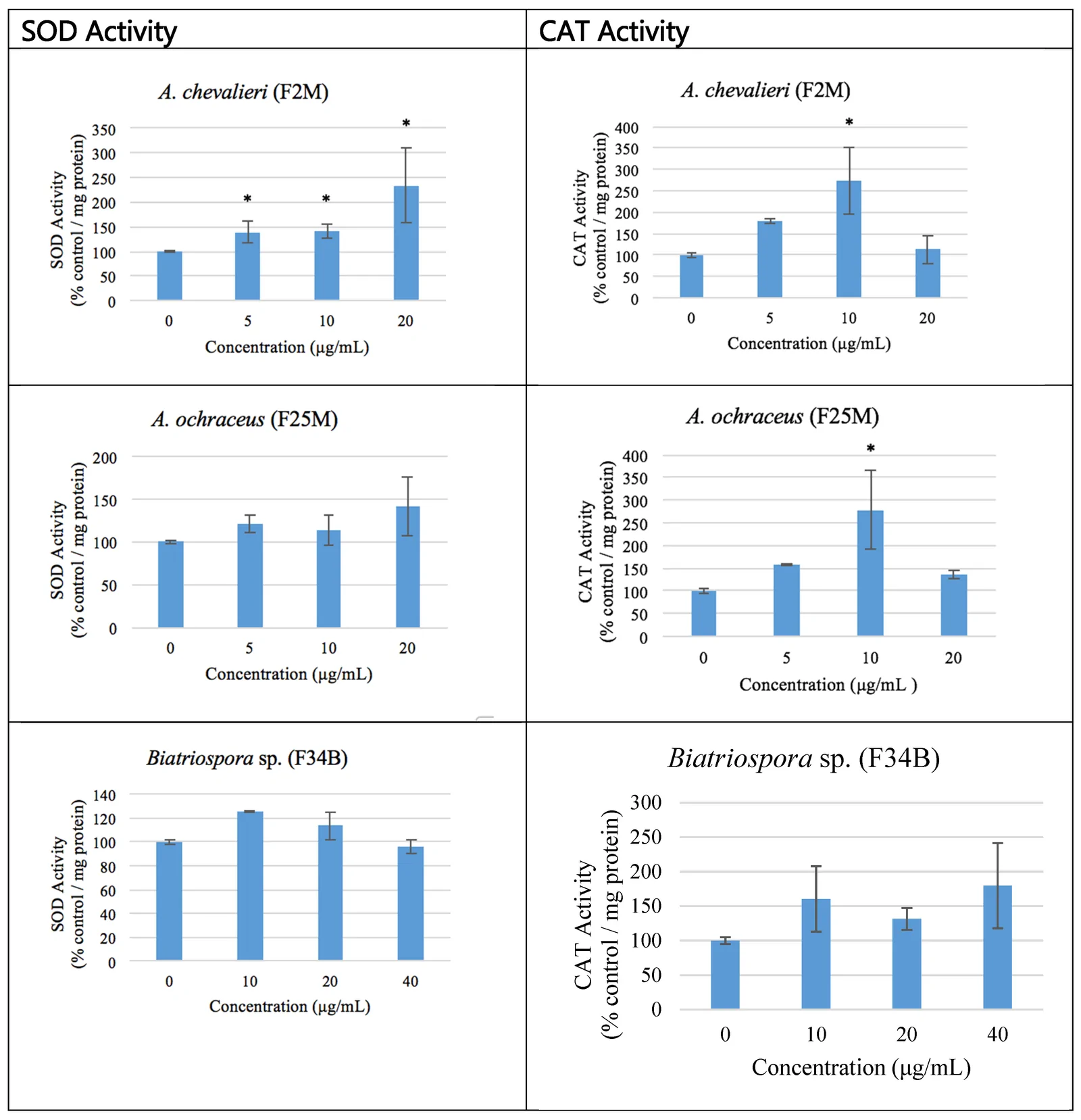

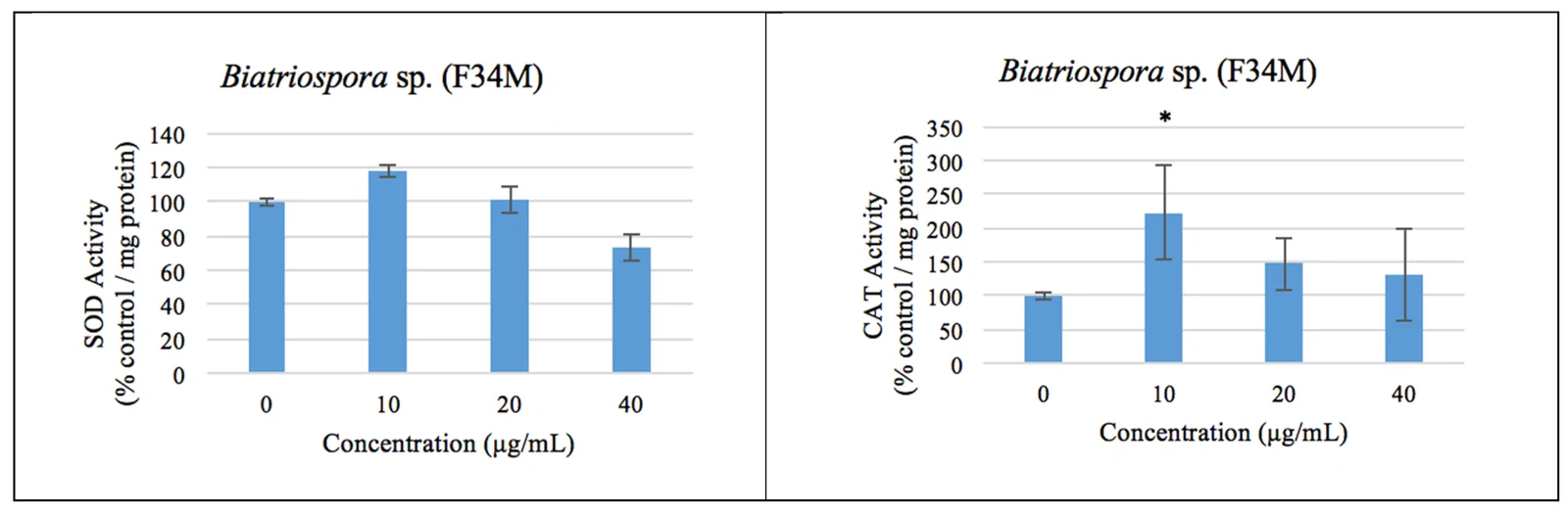
Superoxide dismutase (SOD) and catalase (CAT) activities in HepG2 cells. Significance was assessed by one-way ANOVA followed by Dunnett’s test; * *p* < 0.05 (vs. control). Data are presented as mean ± SD of three independent experiments, with each experiment performed in triplicate (*n* = 3).

Superoxide dismutase (SOD) and catalase (CAT) activities were evaluated to investigate whether the cytotoxic effects of the selected fungal extracts were associated with alterations in antioxidant defense responses. The SOD and CAT activities were assessed at three concentrations, corresponding to half of the IC_50_ value, the IC_50_ value, and double the IC_50_ value. There was a significant increase in SOD activity for the extract of *A. chevalieri* (F2M) (*p* < 0.05) (Figure 5). As concentration of the *A. ochraceus* (F25M) extract increased, the SOD activity also increased. Regarding extracts of *Biatriospora* sp. (F34B and F34M), there was a dose-dependent decrease in SOD activity for the concentrations used.

CAT activity peaked at concentration of 10 µg/mL for the *A. chevalieri* (F2M) and *A. ochraceus* (F25M) extracts. While treatment with 10 µg/mL *Biatriospora* sp. (F34M) also resulted in high CAT activity, the level was considerably lower than with *A. chevalieri* (F2M) and *A. ochraceus* (F25M) extracts.

### 2.5. Metabolomic Profiling Using LC-HRMS

All the extracts studied contained the putatively annotated metabolites riboflavin, (*E*)-N-(4-acetamidobutyl)-3-(4-hydroxy-3-methoxyphenyl) prop-2-enamide, isophorone, palmitamide, 9-octadecenamide and 13-docosenamide. In addition to that, the four tentatively assigned metabolites, namely, mannitol, traumatic acid, 6-methylcoumarin and 13-keto-9*Z*,11*E*-octadecadienoic acid, were present in both species of *Aspergillus* (F2M, F25M). A total of forty-two putatively annotated metabolites (Table S3) were recovered in the mycelium extract of *Aspergillus chevalieri* (F2M). They were alkaloids, amino acids, amines, anthraquinones, carboxylic acids, coumarins, fatty amides, ketones, phospholipids, organic compounds, peptides and terpenoids. A concise summary of the most relevant putatively annotated metabolites and their chemical classes is presented in Table 4.

There were a total of forty-eight putatively annotated metabolites (Table S4) that were recovered from the mycelium extract of *Aspergillus ochraceus* (F25M). They were alkaloids, amino acids, aromatic acids, carboxylic acids, coumarins, fatty acids, fatty amides, ketones, lipids, organic compounds, peptides and a sugar alcohol. A concise summary of the most relevant putatively annotated metabolites and their chemical classes is presented in Table 5.

There were twenty-nine putatively annotated metabolites (Table S5) which were obtained from the broth extract of *Biatriospora* sp. (F34B). They were amino acids, aromatic acids, carboxylic acids, coumarins, fatty acids, fatty amides, ketones, organic compounds, peptides, polyketides and terpenoids. A concise summary of the most relevant putatively annotated metabolites and their chemical classes is presented in Table 6.

There were thirty-two putatively annotated metabolites (Table S6) isolated from the mycelium extract of *Biatriospora* sp. (F34M). They belonged to the classes of amino acids, aromatic amines, carboxylic acids, fatty amides, ketones, lipids, peptides, polyketides and terpenoids. Twenty-two tentatively assigned compounds were common to the broth and mycelium extracts of *Biatriospora* sp. (F34B, F34M). However, more carboxylic acids were present in the broth than in the mycelium extract. Citric acid, pimelic acid and pinolenic acid were present in the broth but absent in the mycelium extract. On the other hand, the five dipeptides, Ser-Phe, Thr-Phe, Ile-Leu, Leu-Phe, Ile-Trp, and the two phosphatidylcholines PC (16:0/18:0) and PC (O-18:0/20:4) were notably absent from the broth extract.

## 3. Discussion

Among the fungal extracts tested, the mycelium extracts of *Aspergillus chevalieri* (F2M) and *Aspergillus ochraceus* (F25M) and the mycelium and broth extracts of *Biatriospora* sp. (F34M, F34B) exhibited the strongest cytotoxic effects. The genus *Aspergillus* is a well-known producer of medicinal secondary metabolites with antimicrobial, cytotoxic, anti-inflammatory and antioxidant properties [19,20]. This study indicates that cytotoxic compounds were mostly present in the mycelium extracts of isolates belonging to the *Aspergillus* genus. Cytotoxic and antiviral nitrobenzoyl sesquiterpenoids from the marine fungus *Aspergillus ochraceus* Jcma1F17 were isolated by Fang et al. [19]. A new sequiterpenoid, 6β,9α-dihydroxy-14-*p*-nitrobenzoylcinnamolide, and insulicolide A had significant cytotoxicity against 10 cancer cell lines with IC_50_ values ranging from 1.95 to 6.35 µM [19]. The mycelial extract of *A. ochraceus* (F25M) also demonstrated a low IC_50_ value of 8.775 ± 0.78 µg/mL against HepG2 cells, supporting the anticancer potential of metabolites produced by this species. The deep-sea derived fungus *Aspergillus chevalieri* MCCC M23426, isolated by Lv et al. [20], produced neoechinulin B and D. These compounds showed cytotoxic properties against the gastric cancer cell MKN1, with IC_50_ values of 20.7 and 4.6 µM, respectively. In line with these findings, the *A. chevalieri* (F2M) isolate used in this study demonstrated a promising IC_50_ of 14.27 ± 1.22 µg/mL against HepG2 cells. Additionally, *A. chevalieri* (F2M) also produced the putatively annotated compounds echinulin and neoechinulin A, which may contribute to its observed cytotoxic activity against the three cancer cell lines used (Figure 1, Figure 2 and Figure 3).

Species of the genus *Nigrograna* can be found in diverse habitats, and includes endophytes and saprophytes. Members of this genus are also producers of natural compounds with bioactive properties and biologically derived fuels [21,22]. The endophytic fungus *Biatriospora* sp. CCF 4378, isolated by Stodůlková et al. [23], showed cytotoxicity against the adenocarcinoma HeLa cells and primary human skin fibroblasts. The compounds responsible for this bioactivity were ascomycone B and 6-deoxyfusarubin. The sponge-associated *Biatriospora* sp. in this study also showed potent cytotoxicity against the three cancer cell lines. The bioactive compounds were present both in the mycelium and the broth extract, and have to be further studied.

The four fungal extracts tested have shown cytotoxic activities against FLO-1 and OE33 esophageal adenocarcinoma cancer cells, with low IC_50_ values ranging from 0.38 ± 0.37 to 128.5 ± 1.87 µg/mL. According to the US National Cancer Institute, a crude extract is considered to have an in vitro cytotoxicity of IC_50_ < 20 µg/mL [24], underscoring the cytotoxic potential of the extracts in this study. The extracts exhibited greater cytotoxicity after 24 h than after 48 h against the esophageal cancer cell lines. This time-dependent difference may reflect the reduced stability of the bioactive compounds or adaptive cellular responses. The underlying mechanism needs to be investigated to understand the modes of action of the active compounds. Moreover, fungal extracts reduced the Incucyte-derived image-based spheroid growth measurement at their highest concentrations (200 µg/mL and 66 µg/mL). Spheroids are three-dimensional (3D) multicellular aggregates formed by cancer cells. 3D tumor spheroid models maintain many of the histological features of tumors, are more representative of cancer, and provide a physiologically relevant model for the study of drug resistance, migration and invasion, as compared to monolayer cultures [25,26]. Previous research has shown that a decrease in tumor spheroid growth following treatment is indicative of cytotoxic activity [27,28]. For instance, treatment with the sponge-derived aeroplysinin-1 resulted in decreased spheroid growth and full inhibition at concentrations of 5 µM and 10 µM, respectively [27]. Khmel et al. [28] isolated petromurin C from the marine-sponge-associated fungus *Aspergillus subramanianii* 1901NT-1.40.2. This compound decreased the area of MCF-7 3D spheroids by approximately 30%. In accordance with these studies, the three extracts examined reduced the Incucyte-derived image-based spheroid growth measurement at their highest concentrations, suggesting an inhibitory effect on spheroid growth and/or integrity (Figure 3).

ROS play an important role in biological processes and their production greatly affects the microenvironments of cells. At high concentrations, ROS are responsible for cancer cell apoptosis [29,30]. The mycelium extract of isolate *A. chevalieri* (F2M) increased intracellular ROS levels in a concentration-dependent manner, with a peak observed at 50 µg/mL. Pearson’s correlation test revealed a relationship between the cytotoxicity and ROS levels (r = 0.883, *p*-value = 0.020) of *A. chevalieri* (F2M). These findings suggest that cell death observed at concentrations up to 50 µg/mL may be mediated by elevated ROS production. Moreover, SOD activity increased significantly when treated with *A. chevalieri* (F2M). This might be a protective mechanism of the cell, helping it to cope with the high levels of ROS. A study by Kim and Park [31] has shown that the marine-algae-associated fungus *Aspergillus fumigatus* produced gliotoxin. This compound increased intracellular ROS, which in turn inhibited NF-kB, resulting in apoptosis of HT1080 fibrosarcoma cells. The mycelium extract of *Biatriospora* sp. (F34M) caused decreases in the SOD and CAT activity levels as the extract concentration increased. Pearson’s correlation test revealed a positive correlation between cytotoxicity and ROS levels (r = 0.852, *p*-value = 0.031). The decrease in antioxidant enzymes and the rise in ROS levels might have caused cell death. A study by Zhang et al. [32] showed that the compound libertellenone H, derived from marine arctic fungus *Eutypella* sp. D-1, increased the intracellular ROS levels in PANC-1 and SW1990 cells, leading to apoptosis. This compound also caused a dose-dependent decrease in intracellular levels of the antioxidant glutathione (GSH). Moreover, pre-treatment of SW1990 cells with SOD prevented the libertellenone H-induced cell growth inhibition, intracellular ROS production and apoptosis [32]. The cytotoxic effects of oxalicumone A (POA), obtained from the marine fungus *Penicillium oxalicum*, were investigated on HK-2 cells by Shi et al. [33]. When HK-2 cells were treated with 40 and 80 µM POA, significant decreases were observed in the cellular antioxidant GSH content and SOD activity, as compared to the control. The percentage of cells that showed ROS accumulation also increased. These results suggest that increased ROS levels might be associated with decreased antioxidant levels and cancer cell death, consistent with the observations in this study.

Several classes of tentatively assigned metabolites were recovered from the four marine fungal extracts. They belonged to the classes of alkaloids, amines, amino acids, anthraquinones, carboxylic acids, coumarins, fatty acids, fatty amides, ketones, lipids, organic compounds, peptides, phospholipids and terpenoids. Notably, six putatively annotated metabolites were common to the four extracts. These were riboflavin, (*E*)-N-(4-acetamidobutyl)-3-(4-hydroxy-3-methoxyphenyl) prop-2-enamide, isophorone, palmitamide, 9-octadecenamide and 13-docosenamide. Riboflavin, palmitamide, 9-octadecenamide and 13-docosenamide have previously been reported in fungi [34,35,36], but two other tentatively assigned metabolites, (*E*)-N-(4-acetamidobutyl)-3-(4-hydroxy-3-methoxyphenyl) prop-2-enamide and isophorone, are found mostly in plants [37,38]. El-Gazzar et al. [39] isolated (*Z*)-13-docosenamide from *Penicillium chrysogenum*. This compound inhibited cell viability and the proliferation of hepatocellular carcinoma [39].

The mycelium extract of *A. chevalieri* (F2M) contained interesting putatively annotated compounds that have been reported in the *Aspergillus* genus. Echinulin, neoechinulin A, preechinulin, flavoglaucin, dihydroauroglaucin and diaporthin are secondary metabolites that have been purified and studied for their biological activities [40,41,42,43]. Calado et al. [44] also identified echinulin and neoechinulin A from the crude extract of *Aspergillus chevalieri*, isolated from the seaweed *Halopteris scoparia*. The crude extract showed strong antioxidant activity, collagenase inhibition and photoprotective ability. The tentatively assigned beta-carotene, a photoprotective compound, was found only in extract F2M. Another marine isolate, *Aspergillus chevalieri* TM2-S6, produced the compound flavoglaucin, which stimulated antioxidant response and stimulated cell proliferation of human dermal fibroblast [42]. The teleomorph of *Aspergillus chevalieri*, the marine *Eurotium chevalieri* MUT 2316, was found to produce echinulin, neoechinulin A, physcion, dihydroauroglaucin and flavoglaucin. These showed antifouling properties, as they prevented the growth/adhesion of bacteria, microalgae and tyrosinase activity [45]. The putatively annotated carviolin, citreorosein, endocrocin and fallacinol are also fungal metabolites but they have been reported in other genera [46]. Other natural products that were tentatively identified only in *A. chevalieri* (F2M) were emodin, rheochrysidin (physcion) and betulin. These are commonly found in fungi and plants and are known for their bioactive properties [47,48,49]. Neoechinulins are diketopiperazine indole alkaloids that have demonstrated cytotoxic properties against different tumor cells [20,40]. The two putatively annotated compounds, emodin and rheochrysidin (physcion), are also known to be apoptosis-inducing in tumor cells [50,51]. The presence of these different putatively annotated compounds in *A. chevalieri* (F2M) extract could explain its significant cytotoxic antioxidant properties in HepG2 cells.

Regarding the mycelium extract of *A. ochraceus* (F25M), the tentatively assigned metabolites asperglaucide, asperphenamate, asperversiamide F, norgeamide D, and stephacidin A were obtained. These are known metabolites that are commonly isolated from *Aspergillus* genus [52,53,54]. The amino acid ester asperphanamate was reported as a cytotoxic compound according to Li et al. [54], Pozzato et al. [55] and Subko et al. [56]. The compound stephacidin A was isolated from a marine *Aspergillus ochraceus* WC76466. This compound was cytotoxic to various cancer cell lines including prostate, ovarian, colon, breast and lung cancer cells [57]. The plant metabolites p-coumaric acid, caffeic acid, ferulic acid and sinapic acid have been tentatively assigned in *A. ochraceus* (F25M). These are usually produced in plants as a defense mechanism, according to Liu et al. [58], and they might have the same role in fungi. The mycelium extract of *Biatriospora* sp. contained a higher number of putatively annotated dipeptides than the broth extract. It has been reported that peptides (ribosomal and non-ribosomal) are produced as defense effectors. While some of them are secreted in the environment, others are stored within the fungal cells [59]. The tentatively assigned metabolites carveol, esculetin, epicathechin, sphondin, bisabolol and nerolidol are produced by plants and have been reported to have bioactive properties [60,61,62,63,64,65]. The two tentatively assigned compounds esculetin and nerolidol have properties reported as cytotoxic and might be responsible for the cytotoxicity of *Biatriospora* sp. (F34B, F34M) extracts [61,66,67,68].

## 4. Materials and Methods

### 4.1. Selection of Fungi for Cytotoxic Properties

Ten fungal isolates with demonstrated antimicrobial activity were selected for cytotoxicity screening [69]. These comprised five endophytic fungi isolated from the brown algae *Turbinaria conoides* and *Sargassum portierianum* and five fungal isolates associated with marine sponges *Biemna* sp., *Haliclona* sp. and *Iotrochota* sp. The sponges and algae were identified based on morphological characteristics, using the World Porifera Database and AlgaeBase, respectively [69]. The fungi were previously identified (Table S1) based on analyses of DNA sequence data [10,69]. Isolates were then cultured under liquid fermentation conditions in seawater potato dextrose broth (PDB). The cultivation broth and mycelia were extracted separately with ethyl acetate to obtain crude broth and mycelial extracts. The algae fungal endophytes were *Aspergillus chevalieri* (F2), *Curvularia verruculosa* (F3), *Acremonium* sp. (F12), *Nigrospora oryzae* (F36), and *Aspergillus iizukae* (F37). The sponge-associated fungi were *Paraconiothyrium* sp. (F23), *Aspergillus ochraceus* (F25), *Parengyodontium album* (F27), *Exserohilum rostratum* (F33), and *Biatriospora* sp. (F34) (Table 7). Both their broth and their mycelium extracts were included in order to test whether the cytotoxic compounds were intracellular or secreted in the culture broth.

### 4.2. MTT Cell Viability Assay

The twenty extracts (broth and mycelium extracts) of the ten selected fungi were tested against the human hepatocellular carcinoma (HepG2, American type culture collection (ATCC), Manassas, VA, USA), human non-small cell lung carcinoma (A549, ATCC, Manassas, VA, USA) and human liposarcoma (SW872, ATCC, Manassas, VA, USA) lines, using the methyl thiazolyl diphenyl-tetrazolium bromide (MTT) cell viability assay. The concentrations of extracts tested ranged from 3.125 to 500 µg/mL. Cells were harvested by trypsinization and plated (8 × 10^3^ cells/well) into 96-well plates in 10% fetal bovine serum (FBS) and Dulbecco’s Modified Eagle Medium (DMEM). They were allowed to grow for 24 h at 37 °C in 5% CO_2_. After 24 h, the media was replaced with the diluted extracts in 1% FBS DMEM. After 24 h, the diluted extracts were discarded and 3-(4,5-dimethylthiazol-2-yl)-2,5-diphenyltetrazolium bromide (MTT) was added. Plates were incubated for 1 h at 37 °C in 5% CO_2_ and the MTT was discarded. Formazan crystals were solubilized in DMSO and the absorbance was read at 570 nm using a microplate reader [70]. The negative control was the 1% FBS DMEM with the equivalent concentration of the solvent DMSO while the positive control was etoposide (1–10 µg/mL). Etoposide was used at concentrations of 1–10 µg/mL to determine its IC_50_ under the same experimental conditions as the fungal extracts. The percentage cell viability values were plotted against the extract concentration and the IC_50_ were determined using GraphPad Prism software (Version 8).

### 4.3. Selection of Fungi for Subsequent Studies

Based on the lowest IC_50_ values, three fungal mycelium extracts, from *A. chevalieri* (F2), *A. ochraceus* (F25), *Biatriospora* sp. (F34), as well as the broth extract of *Biatriospora* sp. (F34), were selected for further studies. These were labelled as F2M, F25M, F34M, and F34B.

The four extracts (3 fungal mycelium extracts and 1 broth extract) were tested against the non-malignant mouse fibroblast 3T3-L1, using the MTT assay, and the selectivity index was calculated as the average IC_50_ value of the normal cell line (3T3-L1) divided by the average IC_50_ value of the cancer cell line (HepG2).

The solid media extracts (F2-SF, F25-SF, and F34-SF) [10] of the three fungi *A. chevalieri* (F2), *A. ochraceus* (F25) and *Biatriospora* sp. (F34) were tested against HepG2 cells, using the MTT assay. This was done to determine if there was a significant difference between the cytotoxic properties of the liquid media mycelium extracts and the solid media extracts. As the liquid media extracts had lower IC_50_ values compared to the solid media extracts, they were selected for subsequent studies. They were F2M, F25M, F34M, and F34B.

### 4.4. Determination of Intracellular Reactive Oxygen Species (ROS) Production

Intracellular ROS level in HepG2 cells treated with varying extract concentrations was determined according to Rummun et al. [71]. HepG2 cells (8 × 10^3^ cells/mL) were allowed to attach in 96-well plates for 24 h. The cells were then treated with the different concentrations of extracts for 24 h. After treatment, the cells were washed thrice with 1× phosphate buffered saline (PBS) and incubated with 100 µL of 10 µM DCFH-DA for 30 min. Cells were washed again with PBS before taking the reading. Fluorescence was measured at excitation/emission wavelengths of 485 nm and 528 nm. The results were normalized to the percentage viability of the cells and expressed as percentage of the negative control.

### 4.5. Evaluation of Intrinsic Antioxidant Activities

HepG2 cells (3 × 10^5^ cells/mL) were seeded in 6-well plates and were allowed to adhere overnight. They were then treated with different concentrations of the extracts. After 24 h, the cells were washed three times with ice-cold 1× PBS and lysed. The lysate was collected and the protein concentration of the cell lysates was determined. The activity of the intrinsic enzymes, superoxide dismutase (SOD) and catalase (CAT), was assessed. For SOD activity, 20 µL of protein lysate was added to 10 µL of 50 mM phosphate buffer and 150 µL reagent mix. Cytochrome c reduction was monitored at 550 nm after addition of 20 µL 4 mU xanthine oxidase at 25 °C for 3 min at every 10 s interval. For CAT activity, 10 µL of lysate was added to 240 µL 0.2% H_2_O_2_. The absorbance was monitored at 240 nm every 7 s for 3 min at 25 °C. The SOD and CAT activity levels were expressed as the percentage of control per mg total protein [71].

### 4.6. Esophageal Adenocarcinoma Cell Line and 3D Tumor Spheroid Assays

#### 4.6.1. Cell Culture

The human esophageal adenocarcinoma FLO-1 and OE33 cell lines were grown in Roswell Park Memorial Institute (RPMI-1640) 1640 medium, supplemented with 10% FBS. The cells were grown under standard culture conditions at 37 °C and 5% CO_2_, in a humidified environment.

#### 4.6.2. Cell Proliferation Assay

The cell density was optimized for 384-well plates at 1500 cells/well for OE33 and 2000 cells/well for FLO-1. The plates were incubated for 24 h in a tissue culture incubator before the addition of test extracts. The test samples were added using a Biomek FX liquid handler (Beckman Coulter Inc., Brea, CA, USA), to give 6-point dose responses with final assay concentrations of 200, 60, 20, 6, 2 and 0.6 µg/mL. Staurosporine (1 µM) and sample vehicle were added as controls. The cells’ proliferation was monitored through the Incucyte ZOOM Live Cell imaging instrument (Sartorius) placed within the humidified 37 °C tissue culture incubator. Phase contrast and RFP images (10× magnification) were acquired at each 3 h interval for 72 h and confluence per well was calculated using the Incucyte ZOOM 2018A software.

At the endpoint, cells were fixed by adding an equal volume of 8% paraformaldehyde (#28908, Thermo Scientific, Waltham, MA, USA) to the existing media to achieve a final concentration of 4%, followed by incubation for 30 min at room temperature. Plates were then washed with PBS, and a staining solution containing 0.1% Triton X-100 (for permeabilization), Hoechst 33,342 (2 µg/mL; Invitrogen, Carlsbad, CA, USA), and Phalloidin-594 (1:1000; ab176757, Abcam, Cambridge, UK), prepared in 1% bovine serum albumin (BSA), was added. After incubation for 30 min at room temperature in the dark, plates were washed three times with PBS and sealed.

Imaging was performed using an ImageXpress Micro XL system (Molecular Devices, San Jose, CA, USA). Images were acquired at 10× magnification, with exposure times kept constant across plates and batches to avoid variability in intensity measurements.

#### 4.6.3. Anticancer Activity Screening on 3D Tumor Spheroid Models

FLO-1 cells were plated at a density of 1000 cells/well in a 384-well ultra-low attachment (ULA) in 5% matrigel. The plates were then spun for 10 min at 1000 rpm, with acceleration set to 1 and no brake on deceleration, so as not to disturb the spheroids. Spheroid formation and growth were allowed for 3 days. The selected extracts and fractions were then added at 4 concentrations (200, 60, 20, and 6 µg/mL). The spheroids’ growth and/or shrinkage were monitored in the Incucyte for the following 5 to 6 days. Spheroid growth was monitored using the ‘Largest Brightfield Object Red Integrated Intensity’ parameter, an image-based measurement used as a proxy for spheroid growth and integrity. Each treatment was performed in triplicate wells across three independent experiments (*n* = 9).

### 4.7. Metabolite Profiling of Fungal Extracts

#### 4.7.1. LC-HRMS Analysis of Fungal Extracts

LC-HRMS analyses were performed on the four selected extracts, namely, the mycelium extracts of *Aspergillus chevalieri* (F2M), *Aspergillus ochraceus* (F25M), and *Biatriospora* sp. (F34M), and the broth extract of *Biatriospora* sp. (F34B), which previously had shown interesting cytotoxic activities. Dried extracts dissolved in methanol at concentrations of 1 mg/mL and 5 μL were injected. Chromatographic separation was performed using an Ultimate 3000 UHPLC system (Themo Fisher Scientific Inc., Waltham, MA, USA), coupled to a Q Exactive Plus Orbitrap mass spectrometer equipped with a heated electrospray ionization (HESI) source. Separation was achieved using a reversed-phase Gemini NX-C18 column (3 µm, 150 × 2 mm) maintained at 35 °C. Mobile phase A consisted of HPLC water with 0.2% acetic acid and mobile phase B consisted of acetonitrile with 0.2% acetic acid. The flow rate was set at 0.5 mL/min. The gradient program started with 95% A and 5% B, and initially held for 1 min; this was followed by a gradual increase of B to reach 100% at 12 min, followed by a hold for 3 min, and then the sequence went back to the initial concentration within 16 min and held for 4 min. The total run time was 20 min. Data was acquired in both the positive and negative ionization modes. The HESI parameters were as follows: sheath gas, 55 arbitrary units; auxiliary gas, 10 arbitrary units; spray voltage at 4.00 kV, capillary temperature, 320 °C; S-Lens RF level, 55% and Aux gas heater temperature 300 °C. Full MS scans were acquired in the range of 100 to 1500 *m*/*z*.

#### 4.7.2. Metabolite Profiling

The MS^2^ data files were converted to .mzXML format using the MSConvert software in the ProteoWizard package [72]. The .mzXML files were then processed using the MZmine 2 v32 [73], in accord with Mintsa et al. [74]. Mass detection was performed by keeping the noise level at 3.0E3. The ADAP chromatogram builder was used with the following settings: minimum group size = 4, group intensity threshold = 3000, minimum highest intensity = 4000, *m*/*z* tolerance = 0.01 Da or 20 ppm. ADAP wavelet deconvolution was carried out using the standard settings, and isotopologues were grouped using the following settings: *m*/*z* tolerance = 0.005 Da or 15 ppm and RT tolerance = 0.3 min. Peak alignment followed by gap filling was done with *m*/*z* tolerance = 0.005 Da or 15 ppm. The .mgf and .csv data files were exported for analysis in GNPS (http://gnps.ucsd.edu). The spectra were searched against the GNPS spectral libraries and the matches were filtered, with criteria of a cosine score above 0.65 and at least 6 matched peaks. Manual verification of MS/MS spectra was also conducted to minimize false-positive annotations. The compounds obtained were compared to the PubChem (https://pubchem.ncbi.nlm.nih.gov/) database and known metabolites were reported.

### 4.8. Statistical Analysis

Statistical analyses were performed using IBM SPSS Statistics (Version 27). All of the experiments were conducted using three independent biological replicates, with technical replicates being averaged prior to statistical analysis. IC_50_ values were calculated using GraphPad Prism (Version 8) by nonlinear regression with a four-parameter logistic variable slope dose–response model. Data normality was assessed using the Shapiro–Wilk test. Two-way ANOVA followed by Tukey’s multiple comparisons for post hoc test was used to determine if there was a significant difference between the mean cytotoxicity of the broth and mycelium extracts. One-way ANOVA and Dunnett’s test were used to determine if there were significant differences in the mean cytotoxicity, ROS production, antioxidant activity and area under curve of spheroids between the fungal extracts and the control. Pearson’s correlation test was used to assess the relationship between cytotoxicity and ROS production.

## 5. Conclusions

The marine fungi investigated produce several natural putatively annotated small molecules which might contribute to their cytotoxic activity against a variety of cancer cell lines. The findings reported in this study highlight the potential of marine-derived fungi, as isolated from sponges and brown algae of Mauritius, as promising sources of cytotoxic metabolites requiring purification and subsequent confirmation and mechanistic studies. The selected extracts have shown moderate to strong cytotoxic properties against the HepG2 liver cancer cell line and were able to modulate intracellular ROS level and intrinsic antioxidant enzyme activities. Moreover, the interesting results obtained on the esophageal adenocarcinoma cell line and 3D spheroid models call for further investigation of the pathways controlling cell cycle progression and leading to cell death. The cytotoxic properties were attributed to certain putatively annotated molecules present in the extracts. Purification of these compounds would be interesting and further studies regarding the interactions of the other chemicals present in the extracts are required. Overall, further investigations are required to elucidate the specific bioactive molecules responsible for the observed cytotoxic effects and to clarify their mechanisms of action in the cancer cell lines studied. Such work will be essential to inform subsequent preclinical evaluation. In this context, marine fungi from the Indian Ocean are a highly promising source of cytotoxic compounds and warrant continued and more detailed exploration.

## Figures and Tables

**Table 1:** Cytotoxicity (IC_50_ µg/mL) of the fungal extracts (from algae) against the three cell lines.

Isolate	Extract	HepG2	A549	SW872
*Aspergillus chevalieri* (F2)	Broth	169.5 ± 21.69	477.5 ± 10.41 ***	230.4 ± 7.64 ***
	Mycelium	14.27 ± 1.22 [SI = 1.70]	29.66 ± 0.50	25.43 ± 2.16
*Curvularia verruculosa* (F3)	Broth	105.8 ± 18.11	316.5 ± 3.63 ***	88.98 ± 9.52
	Mycelium	337.2 ± 36.20	867.4 ± 31.96 ***	497.5 ± 4.20 ***
*Acremonium* sp. (F12)	Broth	455.9 ± 0.28 *	ND	670.2 ± 8.77 ***
	Mycelium	50.93 ± 1.68	66.0 ± 11.80	159.9 ± 9.11 **
*Nigrospora oryzae* (F36)	Broth	265.2 ± 5.21	ND	472.7 ± 9.29 ***
	Mycelium	133.2 ± 7.30	327.0 ± 9.72 ***	188.0 ± 14.75 ***
*Aspergillus iizukae* (F37)	Broth	23.96 ± 9.71	280.0 ± 5.49 *	153.9 ± 5.07 **
	Mycelium	11.66 ± 2.31	114.3 ± 6.58	113.0 ± 10.69 *
Etoposide (Positive control)		5.08 ± 1.11	5.898 ± 2.17	4.280 ± 2.27

Data represent mean IC_50_ values ± SEM of three independent experiments, each performed in triplicate (*n* = 3). Selectivity index (SI) determined as ratio of the IC_50_ non-malignant mouse fibroblast 3T3-L1 to the IC_50_ of the HepG2 cancer cell line. ND = At the highest concentration of extract tested, no cytotoxicity was observed. Asterisks represent significant differences between extracts and etoposide (positive control): * *p* < 0.05, ** *p* < 0.01, *** *p* < 0.001.

**Table 2:** Cytotoxicity (IC_50_ µg/mL) of fungal extracts (from sponges) against the three cell lines.

Isolate	Extract	HepG2	A549	SW872
*Paraconiothyrium* sp. (F23)	Broth	65.37 ± 19.33	393.3 ± 14.16 ***	63.37 ± 13.98
	Mycelium	106.0 ± 7.59	377.5 ± 12.71 ***	138.9 ± 5.44 **
*Aspergillus ochraceus* (F25)	Broth	222.9 ± 17.63	563.0 ± 9.06 ***	290.0 ± 10.30 ***
	Mycelium	8.775 ± 0.78 [SI = 2.27]	19.86 ± 1.46	11.31 ± 1.18
*Parengyodontium album* (F27)	Broth	1016 ± 5.54 ***	ND	ND
	Mycelium	121.0 ± 13.64	327.2 ± 3.37 ***	177.5 ±10.19 ***
*Exserohilum rostratum* (F33)	Broth	31.16 ± 8.33	124.7 ± 5.56	38.54 ± 4.44
	Mycelium	42.09 ± 11.77	169.6 ± 8.51	59.09 ± 0.91
*Biatriospora* sp. (F34)	Broth	19.07 ± 1.70 [SI = 1.30]	22.85 ± 3.90	19.36 ± 2.36
	Mycelium	18.95 ± 1.49 [SI = 1.53]	11.31 ± 4.75	18.01 ± 1.96
Etoposide (Positive control)		5.08 ± 1.11	5.898 ± 2.17	4.280 ± 2.27

Data represent mean IC_50_ values ± SEM of three independent experiments, each performed in triplicate (*n* = 3). Selectivity index (SI) determined as ratio of the IC_50_ non-malignant mouse fibroblast 3T3-L1 to the IC_50_ of the HepG2 cancer cell line. ND = At the highest concentration of extract tested, no cytotoxicity was observed. Asterisks represent significant differences between extracts and etoposide (positive control): ** *p* < 0.01, *** *p* < 0.001.

**Table 3:** Cytotoxicity against esophageal cancer cell lines after 24 h and 48 h.

Esophageal Cancer Cell	Time	*A. chevalieri* (F2M)	*A. ochraceus* (F25M)	*Biatriospora* sp. (F34B)	*Biatriospora* sp. (F34M)
FLO-1	24 h	12.41 ± 1.67	2.49 ± 0.60	10.47 ± 0.80	2.84 ± 3.39
	48 h	28.16 ± 27.90	7.14 ± 3.14	128.5 ± 1.87	13.22 ± 5.52
OE33	24 h	0.53 ± 0.22	0.75 ± 0.33	0.38 ± 0.37	0.53 ± 0.06
	48 h	6.12 ± 10.70	3.14 ± 4.10	4.62 ± 3.53	3.86 ± 1.07

Data are presented as mean IC_50_ values ± SD of three independent experiments, with each experiment performed in triplicate (*n* = 3).

**Table 4:** Summary of putatively annotated metabolites in the *Aspergillus chevalieri* (F2M) mycelium extract.

RT (s)	SpecMZ (*m*/*z*)	Molecular Formula	Main Diagnostic MS/MS Fragments	Adduct Type	Precursor *m*/*z*	Cosine Score	Ion Mode	Compound Name	Class
429.77	301.0703	C_16_H_12_O_6_	215.03, 240.04, 257.04, 286.05	[M+H]^+^	301.071	0.95	+	Fallacinol	Anthraquinone
431.90	299.0550	C_16_H_12_O_6_	227.03, 239.03, 256.04	[M−H]^−^	299.056	0.92	−	Carviolin	Anthraquinone
458.37	326.1858	C_19_H_23_N_3_O_2_	130.06, 198.13, 258.12, 270.12	[M+H]^+^	326.186	0.84	+	(3*S*,6*S*)-3-methyl-6-{[2-(2-methyl-3-buten-2-yl)-1H-indol-3-yl]methyl}-2,5-piperazinedione (Preechinulin)	2,5-diketopiperazine
460.69	324.1699	C_19_H_21_N_3_O_2_	69.07, 185.07, 256.11, 268.11, 324.17	[M+H]^+^	324.171	0.96	+	(3*S*,6*Z*)-3-methyl-6-[[2-(2-methylbut-3-en-2-yl)-1H-indol-3-yl]methylidene]piperazine-2,5-dione (Neoechinulin A)	Indole diketopiperazine alkaloid
460.80	251.0911	C_13_H_14_O_5_	135.04, 149.06, 177.05, 191.03, 233.08	[M+H]^+^	251.091	0.74	+	8-hydroxy-3-[(2*S*)-2-hydroxypropyl]-6-methoxyisochromen-1-one (Diaporthin)	Organic compounds
485.39	285.0396	C_15_H_10_O_6_	211.04, 241.05, 257.05, 285.04	[M−H]^−^	285.04	0.87	−	Citreorosein	Trihydroxyanthraquinone
544.38	313.0344	C_16_H_10_O_7_	225.06, 241.05, 269.05, 313.03	[M−H]^−^	313.035	0.89	−	Endocrocin	Trihydroxyanthraquinone
546.18	285.0751	C_16_H_12_O_5_	242.06, 270.05, 285.07	[M+H]^+^	285.077	0.96	+	Rheochrysidin (Physcione)	Dihydroxyanthraquinone
610.56	269.0447	C_15_H_10_O_5_	197.06, 225.06, 241.05, 269.05	[M−H]^−^	269.046	0.96	−	Emodin	Trihydroxyanthraquinone
740.20	301.1794	C_19_H_24_O_3_	161.06, 189.05, 245.12, 301.18	[M+H]^+^	301.18	0.91	+	Dihydroauroglaucin	Hydroquinone
778.05	305.2103	C_19_H_28_O_3_	161.06, 165.05, 249.15	[M+H]^+^	305.211	0.93	+	2-heptyl-3,6-dihydroxy-5-(3-methylbut-2-enyl) benzaldehyde (Flavoglaucin)	Hydroquinone
782.10	462.3107	C_29_H_39_N_3_O_2_	130.06, 198.13, 210.13, 266.19, 270.12, 338.19, 406.25	[M+H]^+^	462.311	0.86	+	Echinulin	Indole alkaloid
867.30	425.3774	C_30_H_50_O_2_	69.07, 81.07, 95.09, 137.13, 425.38	[M+H−H_2_O]^+^	425.378	0.72	+	Betulin	Triterpenoid

All compounds listed were assigned Level 3 (putatively characterized compound classes) according to the Metabolomics Standards Initiative (MSI) metabolite identification guidelines.

**Table 5:** Summary of putatively annotated metabolites in the *Aspergillus ochraceus* (F25M) mycelium extract.

RT (s)	SpecMZ (*m*/*z*)	Molecular Formula	Main Diagnostic MS/MS Fragments	Adduct Type	Precursor *m*/*z*	Cosine Score	Ion Mode	Compound Name	Class
370.72	223.0598	C_11_H_12_O_5_	93.03, 121.03, 149.02, 164.05, 193.01, 208.04	[M−H]^−^	223.061	0.79	−	Sinapic acid	Hydrocinnamic acid
370.73	163.0386	C_9_H_8_O_4_	79.05, 89.04, 107.05, 117.03, 135.04	[M+H−H_2_O]^+^	163.039	0.92	+	Caffeic acid	Hydrocinnamic acid
373.10	177.0542	C_10_H_10_O_4_	89.04, 117.03, 145.03, 177.05	[M+H−H_2_O]^+^	177.055	0.98	+	Ferulic acid	Hydrocinnamic acid
382.41	147.0437	C_9_H_8_O_3_	91.05, 119.05, 147.04	[M+H−H_2_O]^+^	147.044	0.96	+	p-coumaric acid	Hydrocinnamic acid
519.28	466.2319	C_26_H_31_N_3_O_5_	165.06, 170.07, 212.07, 379.15, 397.16	[M+H]^+^	466.233	0.74	+	Norgeamide D	Pyrroloindole
528.89	432.2262	C_26_H_29_N_3_O_3_	137.06, 165.07, 348.13, 363.16, 387.23, 417.20, 432.23	[M+H]^+^	432.226	0.92	+	Stephacidin A	Prenylated indole alkaloid
582.54	434.2418	C_26_H_31_N_3_O_3_	197.08, 212.11, 280.17, 366.18	[M+H]^+^	434.244	0.81	+	Pyrrolo[1,2-a]pyrazine-1,4-dione, 3-[[7-(1,1-dimethyl-2-propen-1-yl)-2,8-dihydro-2,2-dimethylpyrano[3,2-f]indol-6-yl]methyl]hexahydro- (Asperversiamide F)	Prenylated indole alkaloid
596.39	467.1928	C_27_H_28_N_2_O_4_	105.03, 407.17, 467.19	[M+Na]^+^	467.194	0.76	+	Benzenepropanamide, N-[2-(acetyloxy)-1-(phenylmethyl)ethyl]-alpha-(benzoylamino)- (Asperglaucide)	Organic compound
653.98	507.2265	C_32_H_30_N_2_O_4_	105.03, 117,07, 224.11, 238.12, 256.13	[M+H]^+^	507.227	0.85	+	Asperphenamate	Carboxylic ester

All compounds listed were assigned Level 3 (putatively characterized compound classes) according to the Metabolomics Standards Initiative (MSI) metabolite identification guidelines.

**Table 6:** Summary of putatively annotated metabolites in the *Biatriospora* sp. (F34B, F34M) broth and mycelium extract.

Sample Source	RT (s)	SpecMZ (*m*/*z*)	Molecular Formula	Main Diagnostic MS/MS Fragments	Adduct Type	Precursor *m*/*z*	Cosine Score	Ion Mode	Compound Name	Class
*Biatriospora* sp. (F34B)	303.73	177.0177	C_9_H_6_O_4_	105.03, 133.03, 177.02	[M−H]^−^	177.018	0.93	−	Esculetin	Hydroxycoumarin
*Biatriospora* sp. (F34B)	473.83	135.1166	C_10_H_16_O	91.05, 93.07, 107.09	[M+H−H_2_O]^+^	135.117	0.96	+	Carveol	Monoterpenoid
*Biatriospora* sp. (F34B, F34M)	414.26 (F34B), 377.40 (F34M)	579.1491 (F34B), 579.1495 (F34M)	C_15_H_14_O_6_	245.08, 289.07	[2M−H]^−^	579.151	0.72	−	Epicatechin	Catechin
*Biatriospora* sp. (F34B, F34M)	378.72 (F34B), 379.57 (F34M)	217.0493 (F34B), 217.0492 (F34M)	C_12_H_8_O_4_	161.06, 189.05, 217.05	[M+H]^+^	217.05	0.78	+	Sphondin	Furanocoumarin
*Biatriospora* sp. (F34B, F34M)	705.98 (F34B), 705.11 (F34M)	205.1948 (F34B), 205.1947 (F34M)	C_15_H_26_O	81.07, 93.07, 107.09, 121.10, 149.13	[M+H−H_2_O]^+^	205. 195	0.98	+	Bisabolol	Sesquiterpenoid
*Biatriospora* sp. (F34B, F34M)	720.96 (F34B), 722.05 (F34M)	205.1947 (F34B), 205.1945 (F34M)	C_15_H_26_O	81.07, 93.07, 107.09, 121.10, 149.13, 205.19	[M+H−H_2_O]^+^	205.195	0.99	+	Nerolidol	Farnesane sesquiterpenoid

All compounds listed were assigned Level 3 (putatively characterized compound classes) according to the Metabolomics Standards Initiative (MSI) metabolite identification guidelines.

**Table 7:** Identity of the ten isolates selected for cytotoxic studies.

Isolate	Species name	Host
F2	*Aspergillus chevalieri*	*Turbinaria conoides*
F3	*Curvularia verruculosa*	*Sargassum portierianum*
F12	*Acremonium* sp.	*T. conoides*
F23	*Paraconiothyrium* sp.	*Biemna* sp.
F25	*Aspergillus ochraceus*	*Iotrochota* sp.
F27	*Parengyodontium album*	*Biemna* sp.
F33	*Exserohilum rostratum*	*Haliclona* sp.
F34	*Biatriospora* sp.	*Biemna* sp.
F36	*Nigrospora oryzae*	*S. portierianum*
F37	*Aspergillus iizukae*	*S. portierianum*

## Data Availability

The original contributions presented in this study are included in the article. Further inquiries can be directed to the corresponding author. The New Drug Discovery and Evaluation Center for international cooperation and disciplinary innovation (“111 Center”) at Zunyi Medical University is also acknowledged.

## Supplementary Materials

The following supporting information can be downloaded at: https://www.mdpi.com/article/10.3390/md24080289/s1. Table S1: GenBank accession numbers of fungal isolates; Table S2: Cytotoxicity (IC_50_ µg/mL) of selected fungal extracts against non-malignant mouse fibroblast 3T3-L1; Table S3: Tentative identification of compounds from *Aspergillus chevalieri* (F2M) mycelium extract; Table S4: Tentative identification of compounds from *Aspergillus ochraceus* (F25M) mycelium extract; Table S5: Tentative identification of compounds from *Biatriospora* sp. (F34B) broth extract; Table S6: Tentative identification of compounds from *Biatriospora* sp. (F34M) mycelium extract; Figure S1: (a) Total ion chromatogram (TIC); (b) Extracted ion chromatogram (XIC); (c) High resolution mass spectrum (MS1); and (d) High-resolution tandem mass spectrum (HRMS/MS) of the putatively annotated compound echinulin from *Aspergillus chevalieri* (F2M); Figure S2: (a) Total ion chromatogram (TIC); (b) Extracted ion chromatogram (XIC); (c) High resolution mass spectrum (MS1); and (d) High-resolution tandem mass spectrum (HRMS/MS) of the putatively annotated compound stephacidin A from *Aspergillus ochraceus* (F25M); Figure S3: (a) Total ion chromatogram (TIC); (b) Extracted ion chromatogram (XIC); (c) High resolution mass spectrum (MS1); and (d) High-resolution tandem mass spectrum (HRMS/MS) of the putatively annotated compound esculetin from *Biatriospora* sp. (F34B).
