# Supplementary material for: Cytotoxic Potential of Marine-Derived Fungi Isolated from Sponges and Brown Algae of Mauritius
Source: Mar Drugs. 2026 Aug 21;24(8):289. doi: 10.3390/md24080289 (PMC13514993; doi:10.3390/md24080289)
Supplement: Supplementary file 1 [file marinedrugs-24-00289-s001.zip › marinedrugs-4426414-supplementary.pdf]

Table S1. GenBank accession numbers of fungal isolates.

Isolate	Identity	ITS	LSU	TUB2	CaM	ACT
F2	*Aspergillus chevalieri*	MW187734	OQ754174	OQ793591	OQ923334	
F3	*Curvularia verruculosa*	MW187735				
F12	*Acremonium sp.*	MT799845				
F23	*Paraconiothyrium* sp.	MW187748				
F25	*Aspergillus ochraceus*	MW187750	OQ754175	OQ925676		
F27	*Parengyodontium album*	MW187752				
F33	*Exserohilum rostratum*	MW187757	OQ754176		OQ933786	OQ933784
F34	*Biatriospora* sp.	MW187758	OQ754178	OQ930233		OQ933785
F36	*Nigrospora oryzae*	MW187760				
F37	*Aspergillus iizukae*	MW187761	OQ754177	OQ941652		

Table S2. Cytotoxicity (IC_{50} µg/mL) of selected fungal extracts against non-malignant mouse fibroblast 3T3-L1.

Isolate	Extract	IC_{50} (µg/mL)	Dose-response curve

Aspergillus chevalieri (F2)	Mycelium	24.24 ± 2.421	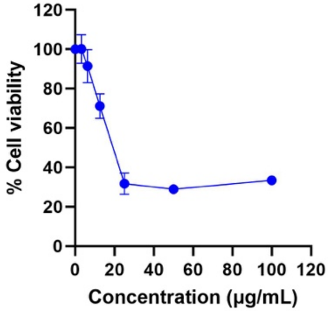
Aspergillus ochraceus (F25)	Mycelium	19.95 ± 1.080	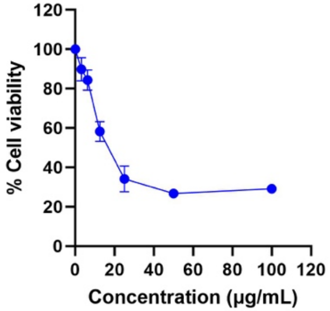
Biatriospora sp. (F34)	Broth	24.73 ± 1.267	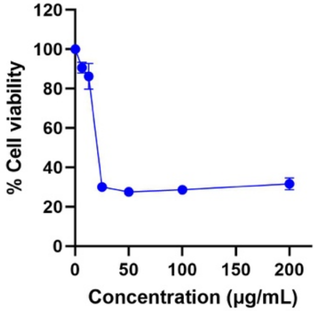

	Mycelium	28.94 ± 2.039	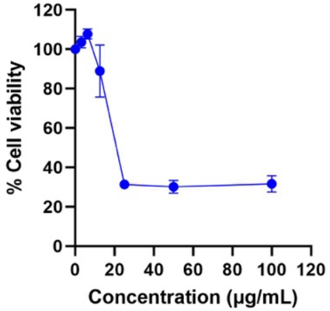

Table S3. Tentative identification of compounds from *Aspergillus chevalieri* (F2M) mycelium extract.

	RT (sec)	SpecMZ (*m/z*)	Molecular Formula	Cosine	MZError PPM	Ion mode	Compound Name	Class
1	39.63	170.0810	$C_8H_{11}NO_3$	0.85	0	+	Pyridoxine	Hydroxymethylpyridine
2	44.32	181.0712	$C_6H_{14}O_6$	0.89	4	-	Mannitol	Sugar alcohol
3	284.21	318.1804	$C_{17}H_{23}N_3O_3$	0.74	4	+	Ile-Trp	Dipeptide
4	299.99	352.1606	$C_{15}H_{21}N_5O_5$	0.98	3	+	Trans-zeatin-riboside	Nucleoside
5	313.92	377.1447	$C_{17}H_{20}N_4O_6$	0.98	3	+	Riboflavin	d-Ribitol
6	353.66	307.1646	$C_{16}H_{22}N_2O_4$	0.86	0	+	(*E*)-N-(4-acetamidobutyl)-3-(4-hydroxy-3-methoxyphenyl)prop-2-enamide	Hydroxycinnamic acid

7	368.56	245.1280	$C_{14}H_{18}N_2O_3$	0.95	4	+	Phe-Pro	Dipeptide
8	373.56	208.0966	$C_{11}H_{13}NO_3$	0.97	1	+	N-acetyl-L-phenylalanine	N-acetyl amino acid
9	426.74	201.1119	$C_{10}H_{18}O_4$	0.89	5	-	Decanedioic acid/Sebacic acid	Alpha, omega-dicarboxylic acid
10	429.77	301.0703	$C_{16}H_{12}O_6$	0.95	2	+	Fallacinol	Anthraquinone
11	431.90	299.0550	$C_{16}H_{12}O_6$	0.92	3	-	Carviolin	Anthraquinone
12	458.37	326.1858	$C_{19}H_{23}N_3O_2$	0.84	0	+	$C_{19}H_{23}N_3O_2$_(3*S*,6*S*)-3-methyl-6-{[2-(2-methyl-3-buten-2-yl)-1H-indol-3-yl]methyl}-2,5-piperazinedione (Preechinulin)	2,5-diketopiperazine
13	460.69	324.1699	$C_{19}H_{21}N_3O_2$	0.96	2	+	(3*S*,6*Z*)-3-methyl-6-[[2-(2-methylbut-3-en-2-yl)-1H-indol-3-yl]methylidene]piperazine-2,5-dione (Neoechinulin A)	Indole diketopiperazine alkaloid
14	460.80	251.0911	$C_{13}H_{14}O_5$	0.74	0	+	8-hydroxy-3-[(2*S*)-2-hydroxypropyl]-6-methoxyisochromen-1-one (Diaporthin)	Organic compound
15	467.07	229.1433	$C_{12}H_{20}O_4$	0.79	0	+	Traumatic acid	Dicarboxylic acid
16	477.83	139.1113	$C_9H_{14}O$	0.95	5	+	Isophorone	Cyclic ketone
17	478.98	318.2995	$C_{18}H_{39}NO_3$	0.81	1	+	Phytosphingosine	Sphingoid

18	485.39	285.0396	$C_{15}H_{10}O_6$	0.87	1	-	Citreorosein	Trihydroxyanthraquinone
19	485.44	157.0757	$C_{10}H_8N_2$	0.91	1	+	Indole-3-acetonitrile	Acetonitrile
20	536.79	299.0186	$C_{15}H_8O_7$	0.84	1	-	Emodic acid	Trihydroxyanthraquinone
21	544.38	313.0344	$C_{16}H_{10}O_7$	0.89	1	-	Endocrocin	Trihydroxyanthraquinone
22	546.18	285.0751	$C_{16}H_{12}O_5$	0.96	6	+	Rheochrysidin (Physcione)	Dihydroxyanthraquinone
23	577.89	371.3259	$C_{21}H_{42}N_2O_3$	0.90	2	+	Myristamidopropyl betaine	Alpha-amino acid
24	593.68	295.2261	$C_{18}H_{30}O_3$	0.71	2	+	13-keto-9*Z*,11*E*-octadecadienoic acid	Fatty acid
25	610.56	269.0447	$C_{15}H_{10}O_5$	0.96	4	-	Emodin	Trihydroxyanthraquinone
26	624.77	170.0961	$C_{12}H_{11}N$	0.97	0	+	Diphenylamine	Aromatic amine
27	709.26	273.1843	$C_{18}H_{24}O_2$	0.87	2	+	Galaxolidone	Organic compound
28	725.49	161.0592	$C_{10}H_8O_2$	0.84	4	+	6-methylcoumarin	Coumarin
29	730.89	173.0594	$C_{11}H_8O_2$	0.71	3	+	Menadione	1,4-naphthoquinone
30	740.20	301.1794	$C_{19}H_{24}O_3$	0.91	1	+	Dihydroauroglaucin	Hydroquinone
31	772.03	734.5673	$C_{40}H_{81}NO_8P$	0.81	3	+	PC (16:0/16:0)	Phosphatidylcholine
32	778.05	305.2103	$C_{19}H_{28}O_3$	0.93	2	+	2-heptyl-3,6-dihydroxy-5-(3-methylbut-2-enyl) benzaldehyde (Flavoglaucin)	Hydroquinone

33	782.10	462.3107	$C_{29}H_{39}N_3O_2$	0.86	4	+	Echinulin	Indole alkaloid
34	784.09	256.2629	$C_{16}H_{33}NO$	0.98	3	+	Palmitamide	Fatty amide
35	784.09	768.5880	$C_{44}H_{83}NO_7P$	0.79	3	+	PC(O-16:0/20:4)	Phosphatidylcholine
36	797.20	265.2519	$C_{18}H_{35}NO$	0.85	0	+	9-octadecenamide	Fatty amide
37	806.35	734.5671	$C_{40}H_{80}NO_8P$	0.89	1	+	1-stearoyl-2-myristoyl-sn-glycero-3-phosphocholine	Phosphatidylcholine
38	821.39	297.1499	$C_{19}H_{20}O_3$	0.84	3	+	NCGC00386103-01_$C_{19}H_{20}O_3$	Organic compound
39	839.22	299.1641	$C_{19}H_{22}O_3$	0.89	0	+	NCGC00386105-01_$C_{19}H_{22}O_3$_5-hydroxy-6-(3-methyl-2-buten-1-yl)-2-[(1*E*)-1-penten-1-yl]-1-benzofuran-4-carbaldehyde	Organic compound
40	858.48	536.4367	$C_{40}H_{56}$	0.71	0	+	Beta-carotene	Carotene
41	867.30	425.3774	$C_{30}H_{50}O_2$	0.72	1	+	Betulin	Triterpenoid
42	898.93	338.3409	$C_{22}H_{43}NO$	0.87	0	+	13-docosenamide	Fatty amide

Table S4. Tentative identification of compounds from *Aspergillus ochraceus* (F25M) mycelium extract.

	**RT (sec)**	**SpecMZ (*m/z*)**	**Molecular formula**	**Cosine**	**MZError PPM**	**Ion mode**	**Compound Name**	**Class**

1	43.97	181.0709	$C_6H_{14}O_6$	0.95	0	+	D-(-)-Mannitol	Sugar alcohol
2	89.75	191.0203	$C_6H_8O_7$	0.93	1	-	Citric acid	Tricarboxylic acid
3	228.41	220.1185	$C_{10}H_{13}N_5O$	0.89	2	+	Zeatin	Aminopurine
4	233.35	245.1852	$C_{12}H_{24}N_2O_3$	0.99	3	+	Ile-Leu	Dipeptide
5	253.23	304.1646	$C_{16}H_{21}N_3O_3$	0.81	1	+	Val-Trp	Dipeptide
6	279.85	279.1693	$C_{15}H_{22}N_2O_3$	0.94	2	+	Leu-Phe	Dipeptide
7	286.70	318.1800	$C_{17}H_{23}N_3O_3$	0.75	3	+	Ile-Trp	Dipeptide
8	301.54	177.0177	$C_9H_6O_4$	0.90	7	-	Esculetin	Hydroxycoumarin
9	314.31	352.1643	$C_{20}H_{21}N_3O_3$	0.94	1	+	Phe-Trp	Dipeptide
10	314.31	377.1443	$C_{17}H_{20}N_4O_6$	0.94	4	+	Riboflavin	d-Ribitol
11	347.96	360.1065	$C_{18}H_{17}NO_7$	0.90	4	+	(2*S*)-3-(3,4-dihydroxyphenyl)-2-[[(*E*)-3-(3,4-dihydroxyphenyl)prop-2-enoyl]amino]propanoic acid (Trans-clovamide)	Tyrosine derivative

12	353.92	307.1644	$C_{16}H_{22}N_2O_4$	0.94	3	+	(*E*)-N-(4-acetamidobutyl)-3-(4-hydroxy-3-methoxyphenyl)prop-2-enamide	Hydroxycinnamic acid
13	370.72	223.0598	$C_{11}H_{12}O_5$	0.79	5	-	Sinapic acid	Hydrocinnamic acid
14	370.73	163.0386	$C_9H_8O_4$	0.92	2	+	Caffeic acid	Hydroxycinnamic acid
15	371.77	208.0964	$C_{11}H_{13}NO_3$	0.99	2	+	L-phenylalanine, N-acetyl-	N-acetyl amino acid
16	373.10	177.0542	$C_{10}H_{10}O_4$	0.98	5	+	Ferulic acid	Hydroxycinnamic acid
17	375.57	261.1316	$C_{12}H_{22}O_6$	0.92	9	-	9-(2,3-dihydroxypropoxy)-9-oxononanoic acid	Fatty acid
18	382.41	147.0437	$C_9H_8O_3$	0.96	2	+	p-coumaric acid	Hydroxycinnamic acid
19	390.63	243.0864	$C_{12}H_{10}N_4O_2$	0.97	6	+	Lumichrome	Flavin
20	416.69	207.0649	$C_{11}H_{10}O_4$	0.87	0	+	Scoparone	Coumarin
21	422.88	261.1590	$C_{15}H_{20}N_2O_2$	0.84	0	+	cyclo(Phe-Leu)	Peptide
22	465.07	229.1433	$C_{12}H_{20}O_4$	0.80	0	+	Traumatic acid	Dicarboxylic acid

23	477.30	139.1113	$C_9H_{14}O$	0.93	5	+	Isophorone	Cyclic ketone
24	479.72	318.2992	$C_{18}H_{39}NO_3$	0.88	2	+	Phytosphingosine	Sphingoid
25	502.56	229.1432	$C_{12}H_{22}O_4$	0.77	7	-	Dodecanedioic acid	Alpha, omega-dicarboxylic acid
26	508.64	352.2007	$C_{21}H_{25}N_3O_2$	0.93	0	+	Tryprostatin B	Cyclic dipeptide
27	508.64	284.1385	$C_{16}H_{17}N_3O_2$	0.93	1	+	cyclo(D-Trp-L-Pro)	Dipeptide
28	513.43	161.0593	$C_{10}H_8O_2$	0.75	4	+	6-methylcoumarin	Coumarin
29	515.41	275.1997	$C_{18}H_{28}O_3$	0.75	4	+	9-OxoOTrE	Fatty acid
30	519.28	466.2319	$C_{26}H_{31}N_3O_5$	0.74	2	+	Norgeamide D	Pyrroloindole
31	526.13	277.2152	$C_{18}H_{30}O_3$	0.89	2	+	13*S*-hydroxy-9*Z*,11*E*,15*Z*-octadecatrienoic acid	Fatty acid
32	528.89	432.2262	$C_{26}H_{29}N_3O_3$	0.92	11	+	Stephacidin A	Prenylated indole alkaloid
33	563.59	161.0956	$C_{18}H_{28}O_2$	0.88	2	+	9,12-octadecadiynoic acid	Fatty acid
34	582.54	434.2418	$C_{26}H_{31}N_3O_3$	0.81	5	+	Pyrrolo[1,2-a]pyrazine-1,4-dione, 3-[[7-(1,1-dimethyl-2-propen-1-yl)-2,8-dihydro-2,2-dimethylpyrano[3,2-	Prenylated indole alkaloid

							f]indol-6-yl]methyl]hexahydro-(Asperversiamide F)	
35	596.39	467.1928	$C_{27}H_{28}N_2O_4$	0.76	2	+	Benzenepropanamide, N-[2-(acetyloxy)-1-(phenylmethyl)ethyl]-alpha-(benzoylamino)-(Asperglaucide)	Organic compound
36	649.02	277.2153	$C_{18}H_{28}O_2$	0.78	2	+	Stearidonic acid	Fatty acid
37	653.98	507.2265	$C_{32}H_{30}N_2O_4$	0.85	0	+	Asperphenamate	Carboxylic ester
38	667.93	279.2309	$C_{18}H_{32}O_3$	0.94	0	+	9(10)-EpOME (Coronaric acid)	Fatty acid
39	673.69	151.1113	$C_{10}H_{14}O$	0.72	4	+	Carvone	Monoterpenoid
40	684.59	295.2262	$C_{18}H_{30}O_3$	0.80	2	+	13-keto-9*Z*,11*E*-octadecadienoic acid	Fatty acid
41	685.04	277.2158	$C_{18}H_{30}O_3$	0.91	0	+	9*S*-hydroxy-10*E*,12*Z*,15*Z*-octadecatrienoic acid	Fatty acid
42	759.03	300.2888	$C_{18}H_{37}NO_2$	0.93	0	+	Palmitoyl ethanolamide	Ethanolamide
43	784.39	256.2627	$C_{16}H_{33}NO$	0.96	2	+	Palmitamide	Fatty amide
44	797.55	265.2518	$C_{18}H_{35}NO$	0.85	0	+	9-octadecenamide, (*Z*)-	Fatty amide

45	834.35	177.1634	$C_{13}H_{22}O$	0.98	3	+	2-butanone, 4-(2,6,6-trimethyl-2-cyclohexen-1-yl)-	Sesquiterpenoid
46	841.38	279.2315	$C_{18}H_{30}O_2$	0.92	1	+	Linolenic acid	Fatty acid
47	898.41	338.3406	$C_{22}H_{43}NO$	0.87	1	+	13-docosenamide, (*Z*)-	Fatty amide
48	1044.30	782.5666	$C_{42}H_{82}NO_8P$	0.87	1	+	1-hexadecanoyl-2-(9*Z*-octadecenoyl)-sn-glycero-3-phosphocholine	Phosphatidylcholine

Table S5. Tentative identification of compounds from *Biatriospora* sp.(F34B) broth extract.

	RT (sec)	SpecMZ (*m/z*)	Molecular Formula	Cosine	MZError PPM	Ion mode	Compound Name	Class
1	38.65	170.0809	$C_8H_{11}NO_3$	0.86	0	+	Pyridoxine	Hydroxymethylpyridine
2	82.32	191.0203	$C_6H_8O_7$	0.94	1	-	Citric acid	Tricarboxylic acid
3	119.73	282.1188	$C_{11}H_{15}N_5O_4$	0.96	3	+	2'-o-methyladenosine	Adenosines
4	298.86	159.0648	$C_7H_{12}O_4$	0.99	9	-	Pimelic acid	Alpha, omega-dicarboxylic acid
5	303.73	177.0177	$C_9H_6O_4$	0.93	7	-	Esculetin	Hydroxycoumarin
6	334.35	307.1644	$C_{16}H_{22}N_2O_4$	0.94	3	+	(*E*)-N-(4-acetamidobutyl)-3-(4-hydroxy-3-	Hydroxycinnamic acid

							methoxyphenyl)prop-2-enamide	
7	313.60	377.1446	$C_{17}H_{20}N_4O_6$	0.97	3	+	Riboflavin	d-Ribitol
8	335.41	177.0543	$C_{10}H_{10}O_4$	0.97	3	+	Ferulic acid	Ferulic acid
9	349.38	211.14	$C_{11}H_{18}N_2O_2$	0.90	1	+	Cyclo(leucylprolyl)	Homodetic cyclic peptide
10	368.80	167.0336	$C_8H_8O_4$	0.91	2	-	2,4,6-trihydroxyacetophenone	Benzenetriol
11	373.33	208.0965	$C_{11}H_{13}NO_3$	0.87	1	+	N-acetyl-L-phenylalanine	N-acetyl amino acid
12	378.72	217.0493	$C_{12}H_8O_4$	0.78	3	+	Sphondin	Furanocoumarin
13	382.38	245.0922	$C_{13}H_{14}N_2O_3$	0.88	3	-	N-acetyl-D-tryptophan	N-acetyl amino acid
14	383.18	247.1068	$C_{13}H_{14}N_2O_3$	0.96	3	+	DL-N-acetyltryptophan	N-acetyl amino acid
15	414.26	579.1491	$C_{15}H_{14}O_6$	0.71	3	-	Epicatechin	Catechin
16	433.20	201.1119	$C_{10}H_{18}O_4$	0.88	5	-	Decanedioic acid/ Sebacic acid	Alpha, omega-dicarboxylic acid
17	467.41	215.1274	$C_{11}H_{20}O_4$	0.72	7	-	Undecanedioic acid	Alpha, omega-dicarboxylic acid
18	472.98	304.1746	$C_{14}H_{22}O_6$	0.85	0	+	methyl 2-ethyl-4-[(3*R*,4*R*,5*S*)-5-hydroxy-4,5-dimethyl-2-oxooxolan-3-yl]-2-methyl-3-oxobutanoate	Polyketide

19	473.83	135.116 6	$C_{10}H_{16}O$	0.96	3	+	Carveol	Monoterpenoid
20	479.67	139.111 4	$C_9H_{14}O$	0.93	4	+	Isophorone	Cyclic ketone
21	551.19	327.122 3	$C_{36}H_{38}N_4O_8$	0.71	3	+	Coproporphyrin I	Coproporphyrin
22	567.83	203.179 3	$C_{15}H_{24}O$	0.90	1	+	1-methyl-4-methylidene-7-(propan-2-yl)-1,2,3,3a,4,5,6,8a-octahydroazulen-1-ol	Organic compound
23	690.07	161.095 4	$C_{18}H_{30}O_2$	0.83	9	+	Pinolenic acid	Octadecatrienoic acid
24	705.98	205.194 8	$C_{15}H_{26}O$	0.98	1	+	Bisabolol	Sesquiterpenoid
25	720.96	205.194 7	$C_{15}H_{26}O$	0.99	1	+	Nerolidol	Farnesane sesquiterpenoid
26	783.95	256.262 8	$C_{16}H_{33}NO$	0.90	3	+	Palmitamide	Fatty amide
27	796.89	282.278 5	$C_{18}H_{35}NO$	0.88	5	+	9-octadecenamide, (*Z*)-	Fatty amide
28	825.27	338.341 0	$C_{22}H_{43}NO$	0.81	2	+	13-docosenamide, (*Z*)-	Fatty amide
29	955.15	871.569 6	$C_{55}H_{74}N_4O_5$	0.76	1	+	Pheophytin a	Pheophytin

Table S6. Tentative identification of compounds from *Biatriospora* sp. (F34M) mycelium extract.

	RT (sec)	SpecMZ (*m/z*)	Molecular formula	Cosine	MZError PPM	Ion mode	Compound Name	Class
1	79.74	252.1084	$C_{10}H_{13}N_5O_3$	0.98	2	+	2'-deoxyadenosine	Purine
2	96.76	253.1174	$C_{12}H_{16}N_2O_4$	0.71	2	+	Ser-Phe	Dipeptide
3	110.73	267.1332	$C_{13}H_{18}N_2O_4$	0.85	2	+	Thr-Phe	Dipeptide
4	119.73	282.1189	$C_{11}H_{15}N_5O_4$	0.96	3	+	2'-o-methyladenosine	Adenosines
5	254.15	245.1853	$C_{12}H_{24}N_2O_3$	0.97	2	+	Ile-Leu	Dipeptide
6	279.84	279.1693	$C_{15}H_{22}N_2O_3$	0.91	2	+	Leu-Phe	Dipeptide
7	298.52	318.1805	$C_{17}H_{23}N_3O_3$	0.82	1	+	Ile-Trp	Dipeptide
8	313.25	377.1447	$C_{17}H_{20}N_4O_6$	0.96	2	+	Riboflavin	d-Ribitol
9	335.05	307.1643	$C_{16}H_{22}N_2O_4$	0.92	3	+	(*E*)-N-(4-acetamidobutyl)-3-(4-hydroxy-3-methoxyphenyl)prop-2-enamide	Hydrocinnamic acid
10	349.64	211.1435	$C_{11}H_{18}N_2O_2$	0.82	2	+	Cyclo(leucylprolyl)	Homodetic cyclic peptide

11	357.29	177.0543	$C_{10}H_{10}O_4$	0.89	3	+	Ferulic acid	Ferulic acid
12	377.40	579.1495	$C_{15}H_{14}O_6$	0.72	2	-	Epicatechin	Catechin
13	379.57	217.0492	$C_{12}H_8O_4$	0.78	3	+	Sphondin	Furanocoumarin
14	380.54	245.0928	$C_{13}H_{14}N_2O_3$	0.85	0	-	N-acetyltryptophan	N-acetyl amino acid
15	382.79	247.1068	$C_{13}H_{14}N_2O_3$	0.98	3	+	DL-N-acetyltryptophan	N-acetyl amino acid
16	432.32	201.1119	$C_{10}H_{18}O_4$	0.89	5	-	Decanedioic acid|Sebacic acid	Alpha, omega-dicarboxylic acid
17	467.48	215.1274	$C_{11}H_{20}O_4$	0.76	7	-	Undecanedioic acid	Alpha, omega-dicarboxylic acid
18	475.49	285.1329	$C_{14}H_{22}O_6$	0.84	3	-	methyl 2-ethyl-4-[(3*R*,4*R*,5*S*)-5-hydroxy-4,5-dimethyl-2-oxooxolan-3-yl]-2-methyl-3-oxobutanoate	Polyketide
19	479.58	318.2994	$C_{18}H_{39}NO_3$	0.87	1	+	Phytosphingosine	Sphingoid
20	480.09	139.1114	$C_9H_{14}O$	0.93	0	+	Isophorone	Cyclic ketone
21	485.06	151.0387	$C_8H_8O_3$	0.92	8	-	3-hydroxyphenylacetic acid	Monocarboxylic acid
22	624.87	170.0963	$C_{12}H_{11}N$	0.93	1	+	Diphenylamine	Aromatic amine

23	705.11	205.1947	$C_{15}H_{26}O$	0.98	1	+	Bisabolol	Sesquiterpenoid
24	716.05	203.1790	$C_{15}H_{24}O$	0.86	0	+	1-methyl-4-methylidene-7-(propan-2-yl)-1,2,3,3a,4,5,6,8a-octahydroazulen-1-ol	Sesquiterpenoid
25	722.05	205.1945	$C_{15}H_{26}O$	0.99	2	+	Nerolidol	Farnesane sequiterpenoid
26	758.35	300.2888	$C_{18}H_{37}NO_2$	0.91	0	+	Palmitoyl ethanolamide	Ethanolamine
27	784.39	256.2629	$C_{16}H_{33}NO$	0.92	3	+	Palmitamide	Fatty amide
28	797.56	282.2784	$C_{18}H_{35}NO$	0.88	5	+	9-octadecenamide, (*Z*)-	Fatty amide
29	808.76	762.5982	$C_{42}H_{85}NO_8P$	0.81	4	+	PC (16:0/18:0)	Phosphatidylcholine
30	811.86	796.6192	$C_{46}H_{87}NO_7P$	0.77	3	+	PC(O-18:0/20:4)	Phosphatidylcholine
31	946.77	871.5696	$C_{55}H_{74}N_4O_5$	0.75	1	+	Pheophytin a	Pheophytin
32	1044.22	338.3405	$C_{22}H_{43}NO$	0.84	1	+	13-docosenamide, (*Z*)-	Fatty amide

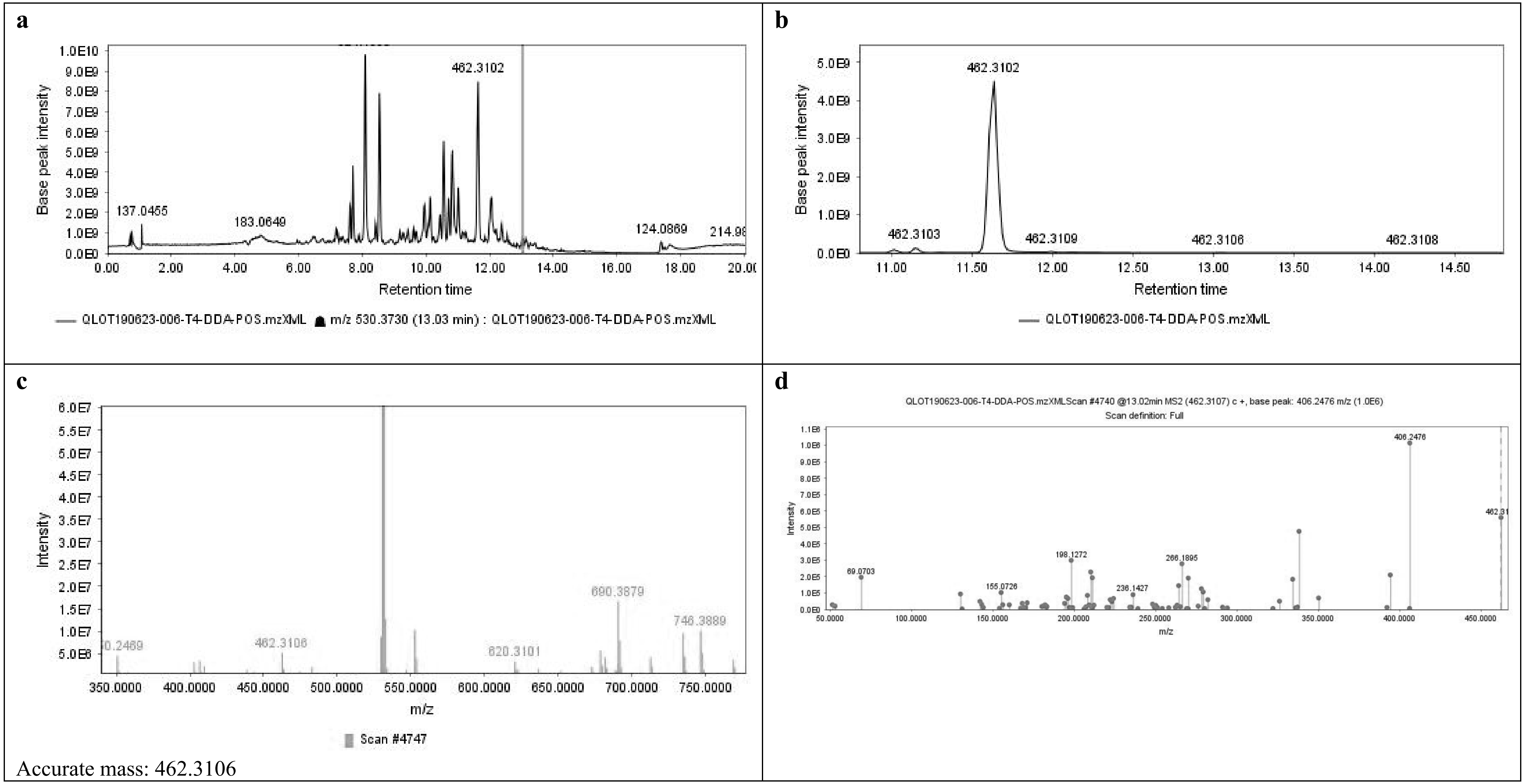

Figure S1. (**a**) Total ion chromatogram (TIC) (**b**) Extracted ion chromatogram (XIC) (**c**) High resolution mass spectrum (MS1) (**d**) High-resolution tandem mass spectrum (HRMS/MS) of the putatively annotated compound echinulin from *Aspergillus chevalieri* (F2M).

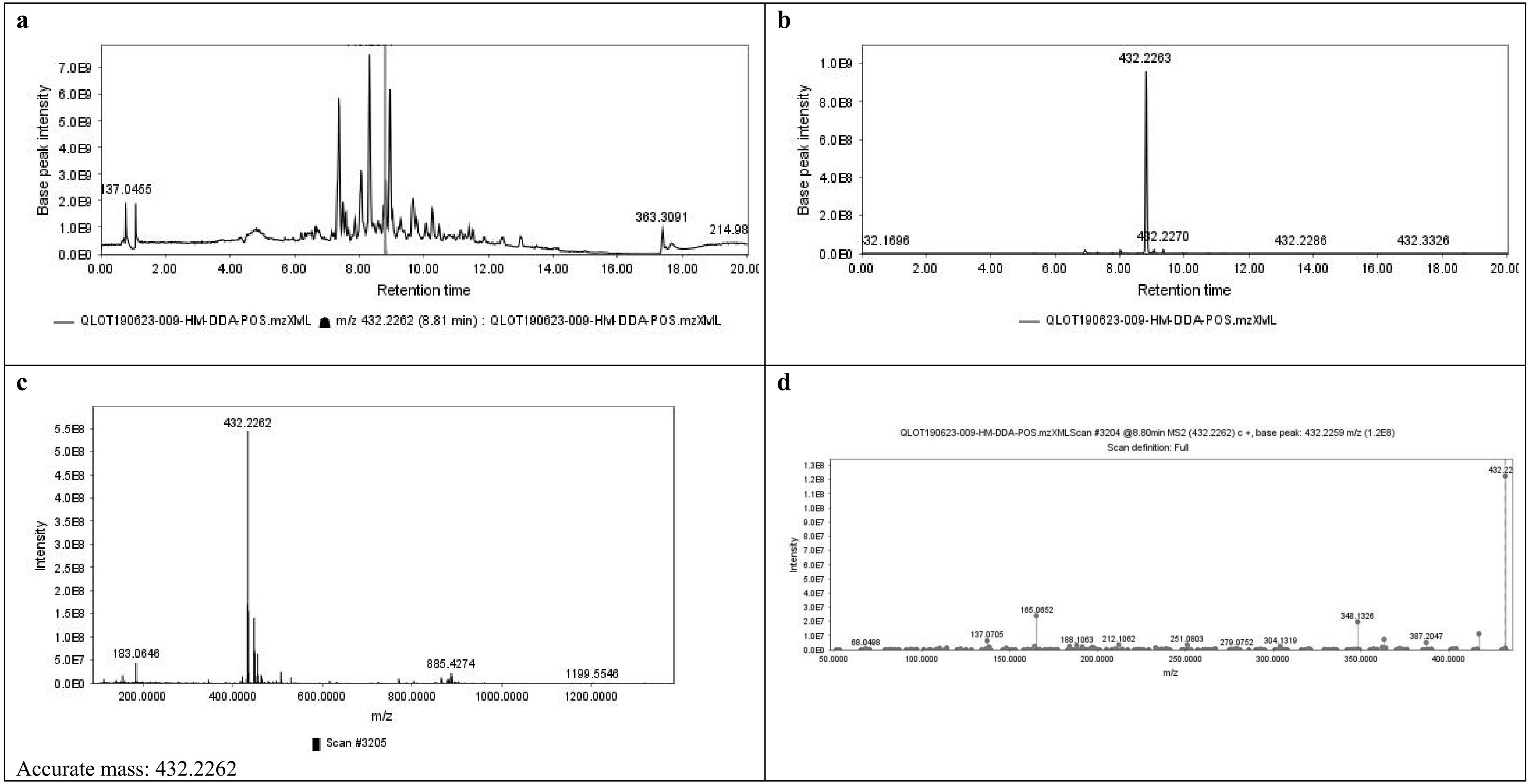

Figure S2. (**a**) Total ion chromatogram (TIC) (**b**) Extracted ion chromatogram (XIC) (**c**) High resolution mass spectrum (MS1) (**d**) High-resolution tandem mass spectrum (HRMS/MS) of the putatively annotated compound stephacidin A from *Aspergillus ochraceus* (F25M).

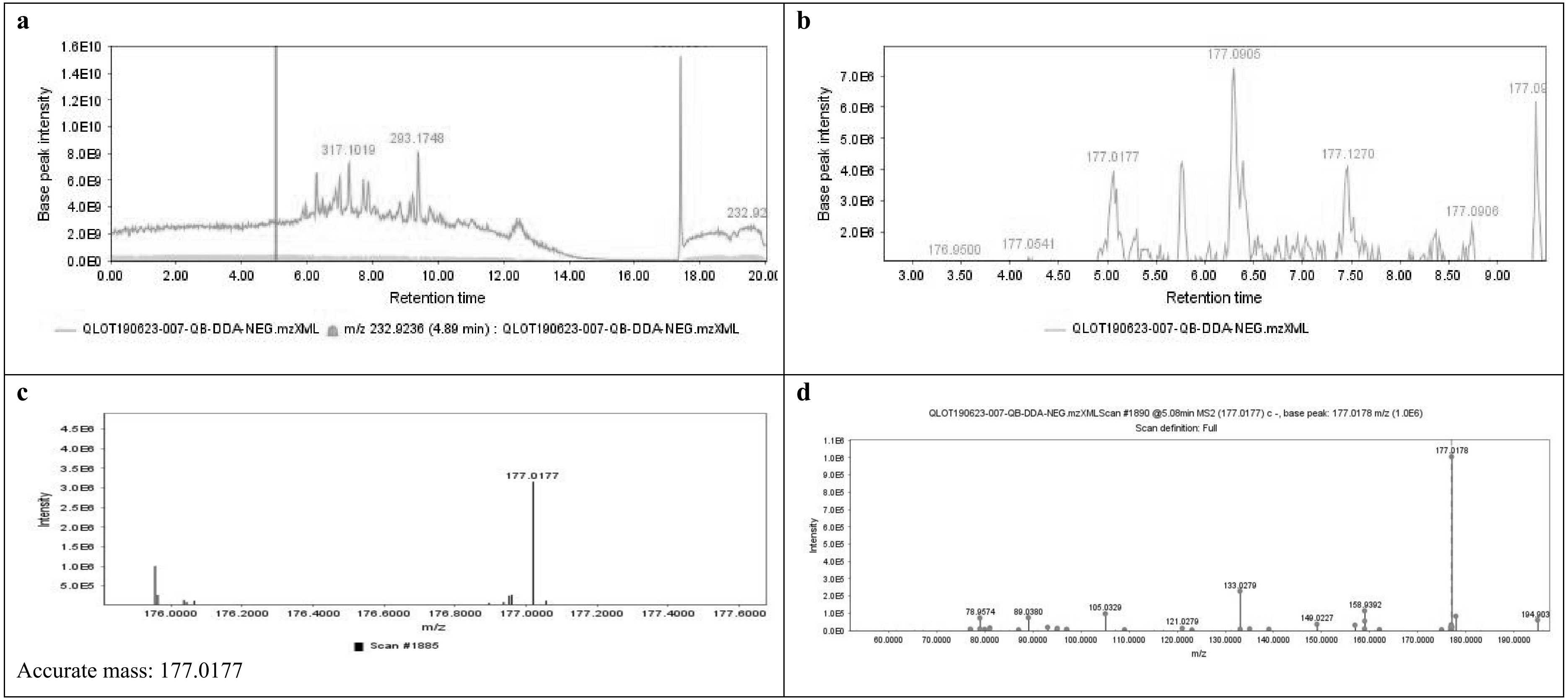

Figure S3. (**a**) Total ion chromatogram (TIC) (**b**) Extracted ion chromatogram (XIC) (**c**) High resolution mass spectrum (MS1) (**d**) High-resolution tandem mass spectrum (HRMS/MS) of the putatively annotated compound esculetin from *Biatriospora* sp. (F34B).
